# Pragmatic, randomized, blinded trial to shorten pharmacologic treatment of newborns with neonatal opioid withdrawal syndrome (NOWS)

**DOI:** 10.1186/s13063-023-07378-x

**Published:** 2023-07-21

**Authors:** Adam Czynski, Abbot Laptook, Abhik Das, Brian Smith, Alan Simon, Rachel Greenberg, Robert Annett, Jeannette Lee, Jessica Snowden, Claudia Pedroza, Barry Lester, Barry Eggleston, Drew Bremer, Elisabeth McGowan

**Affiliations:** 1 Connecticut Children’s Medical Center, Hartford, USA; 2 Brown University/Women & Infants Hospital of Rhode Island, Providence, USA; 3 RTI International, Research Triangle Park, USA; 4 Duke Clinical Research Institute, Durham, USA; 5 National Institutes of Health, Bethesda, USA; 6 University of Mississippi, Oxford, USA; 7 UAMS, Little Rock, USA; 8 University of Texas, Houston, USA; 9 NICHD NRN, Research Triangle Park, USA

**Keywords:** Weaning, Neonatal, Opioid, Withdrawal, NOWS, Morphine, Methadone, Pragmatic, Randomized, Blinded

## Abstract

**Background:**

The incidence of maternal opioid use in the USA has increased substantially since 2000. As a consequence of opioid use during pregnancy, the incidence of neonatal opioid withdrawal syndrome (NOWS) has increased fivefold between 2002 and 2012. Pharmacological therapy is indicated when signs of NOWS cannot be controlled, and the objective of pharmacological therapy is to control NOWS signs. Once pharmacologic therapy has started, there is great variability in strategies to wean infants. An important rationale for studying weaning of pharmacological treatment for NOWS is that weaning represents the longest time interval of drug treatment. Stopping medications too early may not completely treat NOWS symptoms.

**Methods:**

This will be a pragmatic, randomized, blinded trial of opioid weaning to determine whether more rapid weaning, compared to slow wean, will reduce the number of days of opioid treatment in infants receiving morphine or methadone as the primary treatment for NOWS.

**Discussion:**

The proposed study is a pragmatic trial to determine whether a rapid-weaning intervention reduces the number of days of opioid treatment, compared to a slow-weaning intervention, and we powered the proposed study to detect a 2-day difference in the length of treatment. Hospitals will be able to use either morphine or methadone with the knowledge that we may find a positive treatment effect for both, one, or neither drugs.

**Trial registration:**

NCT04214834. Registered January 2, 2020.

**Supplementary Information:**

The online version contains supplementary material available at 10.1186/s13063-023-07378-x.

## Administrative information

Note: the numbers in curly brackets in this protocol refer to SPIRIT checklist item numbers. The order of the items has been modified to group similar items (see http://www.equator-network.org/reporting-guidelines/spirit-2013-statement-defining-standard-protocol-items-for-clinical-trials/).

**Table:** 

Title {1}	Pragmatic, Randomized, Blinded Trial to Shorten Pharmacologic Treatment of Newborns With Neonatal Opioid Withdrawal Syndrome (NOWS)
Trial registration {2a and 2b}.	Trials Registration: NCT04214834; Registered January 2, 2020
Protocol version {3}	Protocol Version 08, July 28,2021
Funding {4}	This research was supported by the National Institutes for Health (NIH) Helping to End Addiction Long-term (HEAL) Initiative® through administrative supplements to NIH grants U24HD095254 and U2COD023375, and NIH grant U24OD024957. Project Officers: Drew Bremer Andrew.Bremer@nih.gov and Carol Blaisdell Carol.Blaisdell@nih.gov , Rockville, MD.
Author details {5a}	Adam Czynski, DO, Connecticut Children’s Medical Center Abbot Laptook, MD, Brown University/Women & Infants Hospital of Rhode Island Abhik Das, PhD, NICHD NRN Brian Smith, MD, Duke Clinical Research Institute Alan Simon, MD, National Institutes of Health Rachel Greenberg, MD, Duke Clinical Research Institute Robert Annett, PhD, University of Mississippi Jeannette Lee, PhD, UAMS Jessica Snowden, MD, UAMS Claudia Pedroza, PhD, University of Texas, Houston Barry Lester PhD, Brown University/Women & Infants Hospital of Rhode Island Barry Eggleston, PhD, RTI International Drew Bremer, MD, NICHD NRN Elisabeth McGowan, MD, Brown University/Women & Infants Hospital of Rhode Island
Name and contact information for the trial sponsor {5b}	National Institutes of Health - Drew Bremer Andrew.Bremer@nih.gov and Carol Blaisdell Carol.Blaisdell@nih.gov
Role of sponsor {5c}	The Eunice Kennedy Shriver National Institute of Child Health and Human Development (NICHD) and NIH Environmental influences on Child Health Outcomes (ECHO) staff are part of the study team and had/will have input into the study design, conduct, analysis, and manuscript drafting. The comments and views of the authors do not necessarily represent the views of the NICHD or the ECHO Program, the National Institutes of Health, the Department of Health and Human Services, or the United States (U.S) government. The NICHD and NIH ECHO staff provided oversight of the project through a cooperative agreement—a grant wherein the funding federal agency is substantially involved in carrying out the research program and federal scientists collaborate with researchers on a joint research project. Only National Institutes of Health staff listed as authors have contributed to this protocol.

## Introduction

### Background and rationale {6a}

The incidence of maternal opioid use in the USA has increased substantially since 2000 [1]. This includes an increase of opioid use during pregnancy including prescription opioids and illicit drugs, as well as a rise in opioid substitution programs for addiction treatment [2]. As a consequence of opioid use during pregnancy, the incidence of neonatal opioid withdrawal syndrome (NOWS) has increased fivefold between 2002 and 2012 [1]. NOWS is a clinical syndrome that reflects signs of withdrawal from opioids in a newborn following in utero exposure. Signs typically occur in the first 5–7 days following birth and reflect dysfunction of the brain, gastrointestinal tract, and autonomic regulation. Simultaneously during this rise in opioid use, the pattern of use has shifted from an inner city, indigent population to a more socioeconomically diverse population. A systematic literature review indicated rural pregnant women have higher rates of polysubstance abuse, as compared to urban pregnant women [3]. The highest incidences of NOWS were reported in the Southeast (i.e., Kentucky, Tennessee, Mississippi, and Alabama) and Northeast (i.e., Maine, New Hampshire, Vermont, Massachusetts, and Rhode Island) United States [4]. This increase in opioid drugs during pregnancy affects neonatal care across the USA. Multiple cross-sectional analyses show that neonatal intensive care unit (NICU) admission rates for NOWS increased from 7 to 27 cases per 1000 admissions and that length of stay increased from 13 to 19 days between 2004 and 2013 [5]. Mean hospital charges for infants discharged with neonatal abstinence syndrome (NAS) increased from $39,400 to $53,400 between 2000 and 2009, and state Medicaid programs bore 78% of these charges [1]. The proportion of neonatal hospital costs due to NAS was estimated to rise from 1.6 to 6.7% between 2004 and 2014 among births covered by Medicaid [6]. Pregnancy complicated by opioid use disorder is associated with high rates of polydrug use, mental health disorders, infectious diseases, poor nutrition, chronic illnesses, and limited social support [7]. Associated risks for newborns beyond NOWS include preterm birth and fetal growth restriction.

Pregnancy represents an opportunity for entry into the healthcare system and initiation of interventions for the mother-infant dyad. However, there are many knowledge gaps in the care of infants with NOWS. The executive summary of a joint workshop by the National Institute of Child Health and Human Development and multiple other partners identified major domains of research priorities on NOWS, including screening and assessment, treatment of NOWS, and transition out of the hospital and follow-up [7].

## Background 

A recent *Journal of Pediatrics* editorial emphasized the rapid rise of NOWS in the USA and provided a framework to target research initiatives and care delivery innovations for infants with NOWS [8]. Specifically, research and quality improvement initiatives should be safe, effective, patient centered, timely, efficient, and equitable. High-quality research is needed to ensure that NOWS care is evidence-based, eliminates non-beneficial practices, and achieves the overarching goals of limiting ongoing opioid exposure for infants, minimizing separation of the mother-infant dyad, and reducing healthcare expenditures. To date, the research community has not rigorously evaluated, through randomized clinical trials, many aspects of NOWS treatment regimens [9].

Signs associated with NOWS reflect dysfunction in several systems: central nervous system (tremors, high-pitched cry, hypertonicity), gastrointestinal (poor feeding, watery, loose stools), and autonomic (hyperthermia). There is widespread acceptance that initial care of infants exposed to opioids in utero should be individualized, supportive, and non-pharmacologic [2]. These measures should include minimizing environmental stimuli (e.g., rooming in [10]), encouraging breast feeding (in the absence of contraindications), and providing sufficient caloric intake. Pharmacological therapy is indicated when signs of NOWS cannot be controlled with non-pharmacological strategies. The objective of pharmacological therapy is to control NOWS signs so that an infant can appropriately bond with her or his mother, tolerate handling and care by healthcare providers, eat effectively with appropriate rest periods to ensure adequate growth, and avoid serious central nervous system dysfunction, such as seizures. Clinical teams traditionally initiate drug treatment when scoring assessments reach a predetermined severity of NOWS signs and include three phases (initiation, stabilization, and weaning). Initiation is the start of drug treatment, and clinical teams progressively increase the dose until the infant achieves stabilization. Stabilization is the interval of time during which the clinical team maintains a drug dose that controls NOWS signs without any indication to further increase the dose. Weaning consists of serial reductions in drug dose and/or lengthening the time interval between doses, and it often begins approximately 48 h after stabilization. NOWS treatment goals should address four domains: (1) support vital neonatal functions (nutrition, appropriate sleep patterns, etc.), (2) promote family bonding, (3) prevent complications (seizures, excessive weight loss, unmanageable irritability), and (4) provide education for the mother-infant dyad and integration into social services to facilitate a smooth transition out of the hospital [7].

Medical professionals do not universally agree on a standard of care for pharmacologically treated NOWS infants [11]. Clinical teams may use different drugs as first-line agents (e.g., morphine, methadone, and buprenorphine) and second-line agents (e.g., phenobarbital, benzodiazepines). At present, morphine is the most commonly used first-line pharmacological treatment for NOWS [12]. Cross-sectional data from the Pediatrix Clinical Data Warehouse showed that the proportion of infants treated with morphine for NOWS increased from 49% in 2004 to 72% in 2013 [5]. Preliminary data from the Advancing clinical trials in neonatal opioid withdrawal syndrome (ACT NOWS) Current Experience, a retrospective chart review conducted among the Institutional Development Awards (IDeA) States Pediatric Clinical Trials Network (ISPCTN) and Neonatal Research Network (NRN), indicated that morphine was the first-line drug for NOWS treatment in approximately 87% of NOWS infants receiving pharmacological treatment. In contrast, clinical teams used methadone in 13% of pharmacologically treated NOWS infants.

Quality improvement methods to standardize NOWS treatment have been successful in reducing the length of treatment and hospital stay among NOWS infants [13]. In contrast, there are limited randomized clinical trials to guide treatment of NOWS infants who require pharmacological therapy. The trials that do exist compared the duration of treatment with morphine and other pharmacological therapies [14–19]; however, these trials were small and collectively included 189 infants treated with morphine and 187 infants treated with phenobarbital, methadone, buprenorphine, or clonidine. There are no clinical trials of different approaches to initiation, stabilization, or the weaning phases of drug therapy. An important rationale for studying weaning of pharmacological treatment for NOWS is that weaning represents the longest time interval of drug treatment. Stopping medications too early may not completely treat NOWS symptoms and may increase the challenges for a family to successfully transition home. Alternatively, excess pharmacological therapy prolongs hospital stay, which increases healthcare utilization and separates the mother-infant dyad.

Kraft et al. summarized the use of morphine and methadone treatment for NOWS [20]. Morphine has a relatively short half-life, and medical professionals administer it every 3 or 4 h. Two principal algorithms for weaning morphine are a percentage reduction (10% of the stabilizing dose every 12-48 h with cessation at 20% of the stabilizing dose) or a fixed reduction (typically decreases of 0.02 mg morphine/dose each day with cessation at approximately 0.02 mg/dose). Although a standard of care for weaning morphine does not exist, all of the referenced clinical trials weaned morphine by 10% reductions of the stabilizing dose [14–19]. However, the research community has not compared weaning by a 10% reduction to a different weaning rate to estimate potential reductions in treatment days without morphine escalation or resumption.

Kraft et al. noted that methadone has a longer half-life than morphine and therefore may be attractive as a therapy due to less frequent administration [20]. However, there is inter-subject pharmacokinetic variability in newborns and children receiving methadone [21, 22]. A pilot study provided important data on the pharmacokinetics of oral methadone for NOWS treatment [22]. Medical professionals have used such a pharmacokinetic-based treatment model to initiate treatment (0.1 mg/kg) with 6-h dosing intervals. If NOWS signs are controlled, they use 12-h dosing intervals to wean the dose from 0.075 to 0.01 mg/kg in six weaning steps, until a final 24-h dose interval. If medical professionals do not readily capture NOWS signs at initiation, they use more frequent dosing intervals (4 to 6 to 8 h) before decreasing doses in 12-h intervals. The Ohio Perinatal Quality Collaborative regimen has used this dosing schedule in a pre-post-intervention study [23].

In contrast to variations for weaning in clinical practice, randomized trials have used 10% reductions of the stabilizing dose. In a single-site trial that compared methadone to morphine, clinical teams weaned both drugs by 10% reductions of the stabilizing dose with 4-h dosing intervals [15]. The most recent, and largest (58 infants in each group), randomized clinical trial of NOWS treatment, performed by Davis et al., was a multi-center trial comparing methadone to morphine [18]. In this trial, medical professionals weaned NOWS infants treated with methadone or morphine by 10% of the stabilization dose every 12 to 48 h with cessation of drug therapy at 20% of the stabilization dose. Administration of methadone alternating with placebo every 4 h and morphine every 4 h facilitated blinding of nursery personnel to the opioid being used. The trial demonstrated that the length of treatment and hospital stay were shorter with methadone, compared to morphine, and these results may prompt a shift from morphine to methadone as the primary opioid to treat NOWS. There are no randomized trials to inform clinicians of potentially better regimens to wean morphine or methadone.

Common outcomes of clinical trials of NOWS treatment are length of treatment, length of hospital stay, and safety outcomes. Although clinically evident brain injury on a neurological exam is not anticipated among infants with NOWS, there is support for abnormalities of neurobehavior [24]. Such information may be important to understand maternal well-being after hospital discharge of a NOWS-treated baby. This is an important domain of NOWS research, and there is a growing recognition that outcomes of NOWS investigations need to broaden to include measures beyond length of treatment and length of hospital stay [7].

The NICU Network Neurobehavioral Scale (NNNS) is a comprehensive evaluation of 12 domains of neurologic and behavioral functioning as well as signs of stress, administered by trained, certified examiners [25]. The research community has used the NNNS to study multiple groups of high-risk infants, including those exposed to drugs in utero (opioids, cocaine) and prematurity [26]. Among 1248 mother-infant dyads enrolled in the Maternal Lifestyle Study, researchers performed NNNS assessments at 1 month *after* hospital discharge [27]. Researchers identified five mutually exclusive neurobehavioral profiles from the 12 neurobehavioral domains by using latent profile analysis. The most atypical profile was characterized by exaggerated scores for arousal, excitability, hypertonicity, quality of movement, and stress abstinence, relative to four other distinct profiles. Researchers have associated this profile with early childhood outcomes, including more externalizing behavior problems, internalizing behavior problems, and total behavior problems at age 3, as well as lower IQ scores after adjustment for gestational age and socioeconomic status [27].

There is a lack of consensus on the effects of prenatal opioid exposure on neurodevelopmental outcomes in early childhood. A recent comprehensive review indicated that there are discrepant findings with respect to the presence or absence of altered neurodevelopment with in utero exposure [28]. This reflects that many studies are small and cannot adjust for potential confounding variables. Potential confounding variables (e.g., prenatal exposures to other substances, nutrition, socioeconomic status, medical complications, poor prenatal care) may all impact early childhood development. Few studies have examined neurodevelopment among infants who develop NOWS, and even less among infants who are pharmacologically treated for NOWS. A retrospective chart review of infants born in 2011–2015 and treated for NOWS with morphine, methadone, or buprenorphine had lower Bayley Scales of Infant Development III at 23 months compared with normative data for the Bayley Scales [29]. Contemporary data on early childhood neurodevelopment of infants with NOWS in the presence or absence of pharmacologic treatment remains a major research gap.

## Preliminary data

### Pilot clinical data

The ISPCTN and the NRN have undertaken a retrospective chart review to inform the design of clinical trials for infants with NOWS (ACT NOWS current experience: Infant exposure and treatment). Investigators reviewed medical records for infants ≥ 36 weeks gestational age and born between July 1, 2016, through June 30, 2017, and mothers medical records, when available, when there was opioid use, determined by maternal history, maternal/infant toxicology screen, or NOWS scoring. Data were collected from 1808 infants at 23 of 28 ISPCTN sites and two of five NRN sites.

The salient findings from the preliminary data of the ACT NOW Current Experience retrospective chart review were:

1. Of infants evaluated for NOWS, medical professionals treated 38.6% with pharmacologic therapy.
2. Of infants treated with pharmacological therapy, the primary medications to control NOWS signs were morphine (86.1%) and methadone (12.9%).

### Site practice for weaning strategies of pharmacological treatment for NOWS

Multiple clinical guidelines from IDeA States Pediatric Clinical Trials Network and the Neonatal Research Network were reviewed to understand the extent of variation in weaning strategies for morphine and methadone. Among centers that use morphine, weaning strategies included reduction by a fixed dose (*n*=2), 10% of the stabilization dose (*n*=6), or 10–20% of the stabilization dose (*n*=3). Among centers that use methadone, weaning strategies included reduction by a fixed dose with changes in frequency of dosing (*n*=2), reductions by 10% of that stabilization dose (*n*=1), and reductions by greater than 10% of the stabilization dose (*n*=3). This review supports a wide range of clinical practices for pharmacologic treatment of NOWS.

### Site practice after cessation of pharmacological treatment for NOWS

Seventeen ISPCTN and NRN sites submitted guidelines and protocols they use to treat infants with NOWS (morphine use: 12 sites, methadone use: 5 sites). In eight of the 17 guidelines, there were specific directives that clinical teams should monitor NOWS infants receiving pharmacological therapy in the hospital for at least 48 h after treatment cessation. In the other nine guidelines, there were no comments on the duration of observation after pharmacological treatment cessation.

### NOWS infants cost of care

Data was obtained from one ISPCTN site to provide an estimate of the *costs* of care for NOWS infants. The cost was $869 per day per infant, which represents the average daily cost among 86 infants born between October 2017 and September 2018. Infants had an average length of hospital stay of 19.4 days, and medical professionals cared for these infants in a family care center that was part of a newborn nursery. The family care center promotes non-pharmacological therapy for newborns exposed to opioids and provides the opportunity for mothers to room in and breast-feed, if there are no contraindications. Costs at hospitals that care for opioid-exposed infants in the NICU may be substantially higher.

## Rationale and summary

Medical professionals pharmacologically treat NOWS infants when non-pharmacological therapy is inadequate to control NOWS signs. The survey data indicate that medical professionals pharmacologically treat a substantial proportion of NOWS infants. There are heterogeneous practices in all aspects of pharmacological treatment (treatment thresholds, initiation, medication type, initial dose, second-line and third-line medications, weaning algorithm, and home therapy). One trial cannot address all the knowledge gaps, and there is limited evidence to guide current clinical management. Clinical trials for this group of patients are challenging for multiple reasons. First, multiple prior randomized trials closed before meeting the projected sample size due to an inability to enroll subjects [15, 17, 18]. Second, hospitals and medical professionals vary in their NOWS treatment practices. Third, although a larger number of hospitals use morphine to treat NOWS, recent clinical trial data suggests that medical professionals may shift to using methadone as the primary opioid for NOWS treatment [18].

Given the uncertainty of the specific opioid medical professionals will use to treat NOWS in the future, the ideal clinical trial would inform clinical practice for the use of either morphine or methadone. To that end, the proposed study is a pragmatic trial to determine whether a rapid-weaning intervention reduces the number of days of opioid treatment, compared to a slow-weaning intervention, and we powered the proposed study to detect a 2-day difference in the length of treatment. Hospitals will be able to use either morphine or methadone with the knowledge that we may find a positive treatment effect for both, one, or neither drugs. We are planning secondary analyses to separately examine the results for each opioid.

The rapidity at which a clinical team can perform weaning with infant tolerance without recurrence of NOWS signs is unknown. In a randomized trial of morphine versus methadone in which clinical teams weaned the drug by 10% of the stabilization dose [18], 48% of morphine-treated and 38% of methadone-treated infants needed dose escalation. With progressive increases in the percent reduction of drug dose, there will, presumably, be an increase in frequency of recurrence of NOWS signs that will mitigate the benefits of more rapid weaning. A 15% reduction of drug dose is large enough to yield important decreases in the length of treatment, which may enable earlier transition out of the hospital and decreasing healthcare costs.

Shortening the weaning phase of NOWS treatment has the potential to impact healthcare costs and minimize the separation of the mother-infant dyad. Opioid use disorder is estimated to occur in 6.5/1000 hospitalizations for infant delivery [30]. This allows an estimate of cost savings for infant’s ≥ 36 weeks gestations:

- Births per year in the USA: ≈ 4,000,000 births
- Percent births ≥ 36 weeks: ≈ 90% × 4 million → 3,600,000
- Opioid exposed: 6.5/1000 deliveries
- Total opioid exposed: 6.5 × 3600 → 23,400
- Opioid exposed receiving pharmacological treatment: 38.6% × 23,400 → 9032
- Cost of care/day: $869 × 9032 → $7,848,808

A treatment reduction of 2.0 days would reduce healthcare costs by more than $15.7 million per year across the USA. Potential cost savings would be even greater for hospitals that care for infants with NOWS in facilities with higher levels of care (e.g., NICU, special care nurseries).

If successful, this clinical trial would achieve the overarching goals of research initiatives for NOWS [8]. Specifically, it would limit ongoing opioid exposure for infants, minimize separation of the mother-infant dyad, and reduce healthcare expenditures.

### Objectives {7}

Primary hypothesis: Among infants receiving an opioid (defined as morphine or methadone) as the primary treatment for NOWS, a rapid-wean intervention will reduce the days of opioid treatment from the first weaning dose to cessation of opioid, compared to a slow-wean intervention.

### Trial design {8}

This will be a superiority trial using a pragmatic, randomized, blinded design comparing a rapid-wean intervention (15% decrements from the stabilization dose) to a slow-wean intervention (10% decrements from the stabilization dose) to determine whether rapid weaning will reduce the number of treatment days among infants receiving morphine or methadone orally as the primary treatment for NOWS. Participating hospitals must use a scoring system to assess for signs of NOWS (original or modified Finnegan Neonatal Abstinence Scoring system, Eat-Sleep, or Console) and provide opioid replacement therapy with either morphine or methadone as the primary drug for treating NOWS. Hospitals may change use of these two opioids during the trial period. We will stratify randomization by hospital. The study protocol will commence after NOWS signs have been controlled with an opioid (stabilization) and weaning of pharmacologic treatment is to be started. At or before each 24-h interval, clinical team members will evaluate and score infants, per hospital practice, for signs of NOWS to determine if the infant will tolerate weaning of the study drug.

- If the infant can tolerate weaning and is in the rapid-wean intervention arm, the clinical team will reduce the study drug by 15% of the stabilization dose. The clinical team will terminate the study drug when the infant can tolerate 25% of the stabilization dose without NOWS signs.
- If the infant can tolerate weaning and is in the slow-wean intervention arm, the clinical team will reduce the study drug by 10% of the stabilization dose. The clinical team will terminate the study drug when the infant can tolerate 20% of the stabilization dose without NOWS signs.
- If infants cannot tolerate weaning in either intervention arm, infants will enter a 12-h period of study protocol guideline that will mandate either weaning or escalating the study drug by the end of the 12-h interval. If the clinical team escalates the study drug, infants will receive opioid using the prior step of the assigned intervention arm.

To maintain blinding of study drug dose during the interventions, the volume of the syringe will be constant and equal the volume of the opioid at stabilization. As the clinical team decreases the study drug during the interventions, the pharmacist will add normal saline to keep a constant syringe volume. Only the pharmacy will be aware of the opioid dose. The use of placebo (normal saline without opioid) in the rapid-wean intervention arm will ensure comparable duration of both weaning interventions.

As part of a pragmatic trial, clinical teams will follow hospital practice for other care practices related to NOWS treatment (type of scoring system, threshold to initiate treatment, duration of stabilization, use of second-line and third-line drugs, rooming in, breast milk, etc.). After study drug cessation, the clinical team will observe infants in the hospital for at least 48 h prior to discharge, which is similar to clinical practice. A trained examiner will administer the NNNS to assess neurobehavioral profiles after infants cease study drug and prior to discharge. Some participating sites may need to train their study staff on the NNNS procedure. The NNNS training requires video recordings of infants sent to the training center at Brown University. Only the trainers at the Brown Center or their trainer designee and site trainees will have access to the video. The video will be deleted from the server once it has been reviewed for training purposes, and training on that video is complete. These infants may or may not be otherwise involved in the protocol. Sites may assess infants who will not enroll in the study, infants who will enroll, or both for this training. Because this training activity will not yield study data, a separate consent form will be used for this training.

At 1 month post-discharge, primary caregivers will complete the Parent-Reported Outcome Measure Information System (PROMIS) Measures, the Maternal Postnatal Attachment Questionnaire (MPAQ), and a caregiver questionnaire. The site research team will contact the primary caregiver(s) to update contact information and/or complete questionnaires when the infant is 6, 12, 18, and 24 months of age. The questionnaires will assess infant wellness, neurobehavioral functioning and development, postnatal attachment and bonding, and caregiver well-being. At 24 months, the infants will be seen during which a certified developmental specialists, blinded to the intervention, will administer the Bayley Scales of Infant and Toddler Development, Fourth Edition (Bayley-4) to assess infant neurodevelopment. The PROMIS Measures and the Brief Infant Toddler Social Emotional Assessment (BITSEA) will also be administered during the 24-month visit along with measures of growth.

## Methods: participants, interventions, and outcomes

### Study setting {9}

The study is being conducted in academic medical centers across the USA. A complete reference list of study cites can be obtained at the following website: https://beta.clinicaltrials.gov/study/NCT04214834.

### Eligibility criteria {10}

#### Eligibility criteria

##### Inclusion criteria

*Hospital level*

1. Hospital provides pharmacologic treatment to at least an average of 12 opioid-exposed infants each year
2. Hospital uses a scoring system to assess for signs of NOWS (original or modified Finnegan Neonatal Abstinence Scoring system, Eat-Sleep or Console)
3. Hospital provides opioid replacement therapy with either morphine or methadone as part of pharmacologic treatment of NOWS

*Infant level*

Infants need to fulfill all of the following criteria:

1. Gestational age ≥ 36 weeks
2. Receiving scheduled pharmacological therapy with morphine or methadone as the primary drug treatment for NOWS secondary to maternal opioid use
3. Tolerating enteral feeds and medications by mouth

##### Exclusion criteria

*Hospital level*

1. Hospitals discharge> 10% of infants from the hospital on opioid replacement therapy on average per year

*Infant level*

Any of the following is an infant level exclusion criterion:

1. Major birth defect (e.g., gastroschisis)
2. Any major surgery (minor surgery [e.g., circumcision, digit ligation, frenulectomy] is not an exclusion)
3. Hypoxic-ischemic encephalopathy
4. Seizures from etiologies other than NOWS
5. Treatment with opioid for reasons other than NOWS
6. Respiratory support (nasal cannula or greater) for > 72 h
7. Planned discharge from the hospital on opioids
8. Use of other opioids (e.g., buprenorphine) as primary drugs for treatment
9. Weaning of morphine or methadone as the primary treatment of NOWS has started

### Who will take informed consent? {26a}

Research staff may reach out to, and obtain consent from, pregnant women and postpartum women of eligible or potentially eligible infants at any of the following times: (a) prior to birth, (b) after birth but prior to the determination of the infant’s eligibility, (c) after birth and after infant’s eligibility has been confirmed. The legal guardian or legally authorized representative may be approached when the mother does not have custody.

#### Pregnant women who are using/used opioids while the infant is in utero

Research personnel may approach pregnant mothers who are using (used) opioids while the infant is in utero*.* Research personnel may use site-specific practices to introduce the study and start the consent process prior to the mother giving birth. Additionally, the informed consent form may be completed (signed) *prior to* the mother giving birth. For those mothers that consent before delivery, the research team will meet with the mother after delivery to obtain written confirmation of her continued willingness to allow her infant to be part of study and for her willingness to be a participant herself.

Eligibility of the infant can only be determined after delivery. The mother is not eligible if the infant is not eligible. The research team will tell all mothers whether or not their infant met eligibility requirements.

#### Postpartum mothers

Research personnel may approach postpartum mothers of potentially eligible infants as well as mothers of infants known to meet the eligibility criteria.

For mothers that sign the consent prior to the infant meeting eligibility requirements, the mothers will be informed of their infants’ eligibility status once that status has been determined.

The time period for approaching pregnant women and postpartum mothers, therefore, includes prenatal clinic visits through completion of stabilization of the infant, but prior to the start of opioid weaning of the infant. The mother is not eligible if the infant is not eligible.

#### General

Research personnel will obtain informed consent from the infant’s parent or legal guardian (legally authorized representative). If there are any concerns regarding the cognitive status of the mother, the site PI or designee will be consulted. If the infant’s mother is cognitively impaired and is unable to provide informed consent to the research study, then an alternative legal guardian may be approached for consent per local guidelines. Sites will follow location-specific requirements for enrollment of wards of the state. If legal guardianship changes, the new legal guardian would be contacted to obtain consent for the study.

#### Infant-only and caregiver-only consents

The mother may opt to allow her infant to be in the study, but not be a participant herself. If the mother agrees to allow the infant to be a participant, but not be a participant herself, then she will sign the infant-only consent. Similarly, if the legal guardian is not the caregiver or does not want to be a participant him/herself, but the legal guardian is willing to allow the infant to be a participant, the legal guardian will sign the infant-only consent. If a caregiver is not the legal guardian of the infant, but the caregiver is willing to answer questions about him/herself, the caregiver will sign the caregiver-only consent.

#### Consents for custody changes

Laws vary by state. Sites should consult with appropriate entities (e.g., local university/hospital legal counsel, local Institutional Review Board (IRB), central IRB (cIRB), study team operational principal investigator, et al.) to ensure the correct consents are signed and new consents obtained as needed.

#### Remote consent

To facilitate the consenting process, due to (a) the ongoing COVID-19 pandemic, (b) the potential for change in guardianship, and (c) the potential for a non-emancipated minor mother reaching legal age of majority, remote consenting will be allowed. When conducting remote consent, all communications will be done via Health Insurance Portability and Accountability Act (HIPAA)-compliant methods such as telephone, personal delivery of documents, US postal service, REDCap, or other compliant electronic platform. The remote consent process will parallel the consent process used for in-person consenting. The only difference will be the method(s) of communication. The study team will ensure that, as with in-person consenting, the participant is given sufficient opportunity to ask questions, is able to understand the nature of this study and what participation entails. The study team will ensure the participant is provided a copy of the final, completed consent, signed by all parties involved, including the research team member who obtained consent and, when applicable, the site investigator. This final, signed consent will be provided via a HIPAA-compliant method or a method that the participant has agreed to in writing. The study team members working on the consenting process will ensure that any participant who is consenting remotely has the authority to consent.

### Additional consent provisions for collection and use of participant data and biological specimens {26b}

N/A; we will not collect biological specimens during the conduct of this study.

## Interventions

### Explanation for the choice of comparators {6b}

N/A the information requested for a list of comparators would be redundant to the information provided within the background and rational.

### Intervention description {11a}

**Fig. 1:**
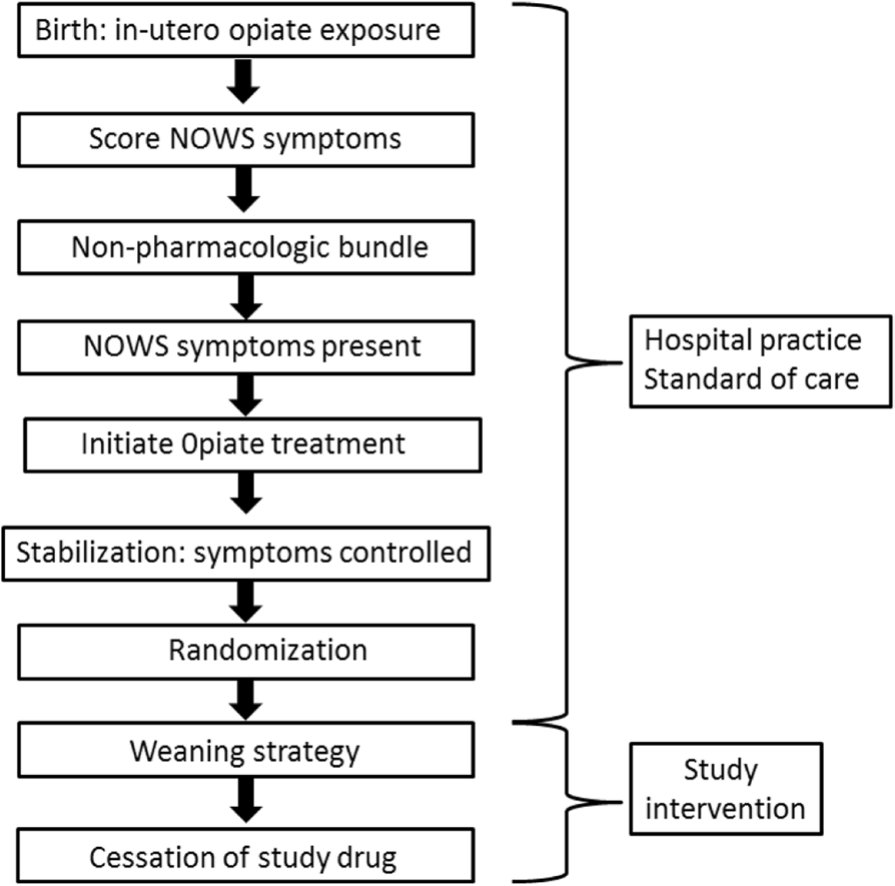
Overview of study timing and intervention

This will be a pragmatic, randomized, blinded trial of opioid weaning to determine whether more rapid weaning, compared to slow wean, will reduce the number of days of opioid treatment in infants receiving morphine or methadone as the primary treatment for NOWS. Figure 1 illustrates when the study interventions will occur during the hospitalization.

Consistent with the pragmatic design, hospitals will use their specific management practices for opioid treatment among NOWS infants after birth and prior to randomization and the start of opioid weaning. This may include the following management practices:

- Location of care of the infant (mother-baby unit, NICU, Pediatric floor etc.).
- Frequency of monitoring of vital signs and use of cardiopulmonary monitors.
- A non-pharmacological bundle to control NOWS signs. We will recommend a standardized bundle, but hospitals will be able to optimize it for their own use.
- Use of breast milk and breast feeding.
- Scoring assessments of NOWS signs.
- Scoring criteria to initiate opioid therapy.
- Choice of opioid (morphine or methadone) as the primary treatment and dosing to initiate pharmacological therapy.
- Initiation and adjustment of dosing of second-line and third-line drugs for NOWS signs (e.g., phenobarbital, clonidine) if NOWS signs are not adequately controlled with an opioid.
- Duration of stabilization whereby the clinical team controls NOWS signs before they initiate opioid weaning.

#### Study intervention

**Fig. 2:**
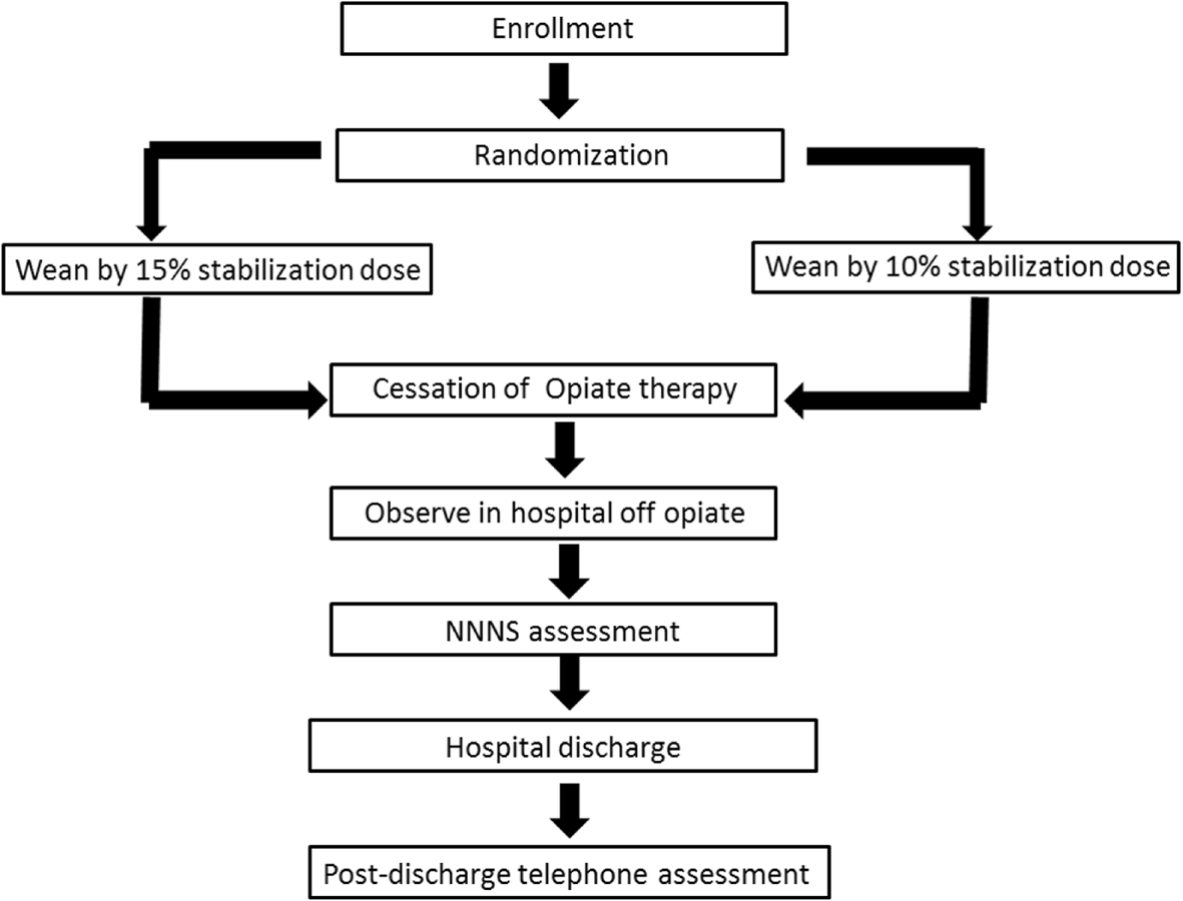
Overview of the study interventions

We will randomize infants to either a rapid-wean intervention arm or a slow-wean intervention arm (Fig. 2; Table 1). Infants in the rapid-wean intervention arm will undergo opioid reduction by 15% of the stabilization dose whenever the clinical team weans the opioid. The clinical team will terminate the opioid when the infant can tolerate 25% of the stabilization dose without NOWS signs. Infants in the slow-wean intervention arm will undergo opioid reduction by 10% of the stabilization dose when the clinical team weans the opioid. The clinical team will terminate the opioid when the infant can tolerate 20% of the stabilization dose without NOWS signs.

**Table 1:** Dose levels of the rapid-wean and slow-wean interventions

**Dose**	**Rapid wean: % of stabilization dose**	**Slow wean: % of stabilization dose**
Stabilization Dose	100	100
Dose level A	85	90
Dose level B	70	80
Dose level C	55	70
Dose level D	40	60
Dose level E	25	50
Dose level F	Placebo	40
Dose level G	Placebo	30
Dose level H	Placebo	20

The research team will distinguish dose levels from study steps for the clinical team and the pharmacy during training in-services. There are eight dose levels for the rapid- and slow-wean intervention arms, each representing the amount of opioid the clinical team will administer. Study steps represent the number of time intervals between different dose levels. If opioid escalation does not occur, the infant will receive eight dose levels in eight study steps. However, if there are escalations, the clinical team will need to repeat dose levels and there will be more study steps than dose levels. The distinction between dose level and study steps is depicted in Table 2.

**Table 2:** Differences between dose level and study steps for study drug escalation

**Steps**	**Rapid-wean intervention**		**Slow-wean intervention**	
	**Dose level**	**% of stabilization dose**	**Dose level**	**% of stabilization dose**
Step 0	Stabilization	100%	Stabilization	100%
Step 1	Dose level A	85%	Dose level A	90%
Step 2	Dose level B	70%	Dose level B	80%
Step 3	Dose level C	55%	Dose level C	70%
***Step 4***	***Dose level B***	70%^*^	***Dose level B***	80%^*^
Step 5	Dose level C	55%	Dose level C	70%
Step 6	Dose level D	40%	Dose level D	60%
Step 7	Dose level E	25%	Dose level E	50%
Step 8	Placebo	Placebo	Dose level F	40%
***Step 9***	***Placebo***	Placebo^*^	***Dose level G***	30%^*^
Step 10	Dose level E	25%	Dose level F	40%
Step 11	Placebo	Placebo	Dose level G	30%
Step 12	Placebo	Placebo	Dose level H	20%

^*^dose level at a given study step was not successfully completed and resulted in an escalation

The asterisks indicate that the dose level at a given study step was not successfully completed and resulted in an escalation. The pharmacy will track dose levels to know where an infant is within a rapid- or slow-wean intervention arm. The clinical team will be blinded to the dose level and will only be aware of the study steps. Both the rapid- and slow-wean intervention arms are depicted to indicate that if each intervention arm has the same number of escalations, the study steps will be identical. This is critical to maintaining the clinical team blinding.

#### Choice of opioid and dose frequency

- The choice of opioid will be per individual hospital practice.
- The dose interval for morphine will be either every 3 or 4 h, per hospital practice.
- The dose interval for methadone will be every 8 or 12 h, per hospital practice.

#### Changes in opioid dose

**Fig. 3:**
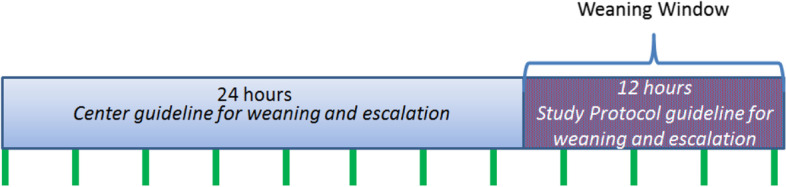
Overview of the time periods used by clinical team to either wean or escalate study drug. A 24-hour period for weaning or escalation, per hospital guidelines (light blue bar). Multiple vertical green lines represent dosing intervals; in this example the infant is receiving an opioid every 3 h. If the opioid dose does not change after 24 h of dosing, the infant enters a 12-h period of study protocol guideline (purple bar) to ensure that hospitals either wean or escalate and do not remain on the same dose. This approach will be applied to both weaning interventions

The following are general considerations for *both* rapid- and slow-wean intervention arms from the first weaning dose to cessation of study drug:

- The clinical team will use hospital-specific tools to determine the severity of NOWS signs (Finnegan; modified Finnegan; Eat, Sleep, Console; etc.).
- The clinical team will assess infants for NOWS signs every 3 or 4 h prior to care times (clinical assessment, vital signs, and feeding).
- The clinical team will use hospital thresholds of NOWS signs (e.g., wean if all Finnegan scores are < 8, escalate for an average of three scores ≥ 8 or two scores ≥ 12) to trigger changes in study drug dose.
  - We will have one exception to hospital thresholds for changes in opioid dose based on a prior randomized trial [31]. Infants with an elevated NOWS score, that would prompt an escalation, should be fed and rescored within 1 hour of the start of the feed. The Clinical team should use the lower of the two scores when evaluating the series of scores for escalation.
- We will provide each hospital’s pharmacy a dosing calculator. After randomization, the pharmacy will input the weaning intervention (wean by 10 or 15% of the stabilization dose), the stabilization dose (mg/kg/day), the infant’s weight, and the dosing interval (every 3, 4, 8, or 12 h) to identify the steps of the intervention arm. The dosing calculator will provide the absolute dose (mg) at each step of the intervention arm.
- Frequency of dose changes for *weaning*:
  - We will encourage clinical teams to wean study drug at least every 24 h.
  - Clinical teams may wean infants at ≤ 24 h of a given dose (< 8 doses when given every 3 h, < 6 doses when given every 4 h, < 3 doses when given every 8 h, and < 2 doses when given every 12 h), per *hospital guideline*.
  - Infants not weaned by 24 h of a given dose will enter a 12-h period of *study protocol guidelines* (Fig. 3). During this 12-h period, the clinical team *must* wean infants who do not meet hospital-specific criteria for escalation. Hospitals do not need to use the total 12-h period to either wean or escalate if the infant meets the criteria prior to 12 h. The research team will use dedicated in-services for all clinical teams of participating hospitals prior to study start on the specifics of the trial intervention including the 12-h study protocol guideline.
  - The clinical team will order, “wean opioid per protocol” to trigger weaning.
- Frequency of dose changes for *escalation*:
  - Clinical teams may escalate the opioid at any time during the study intervention based on hospital guidelines; clinical teams do not need to wait for 24 h of dosing.
  - The clinical team will order, “Escalate opioid per protocol” to initiate escalation.
  - When the clinical team orders opioid escalation, the pharmacy will resume the preceding step of the intervention arm. For example, an infant receiving placebo in the rapid-wean intervention arm will escalate to the last opioid dose (25% of the stabilization dose). The clinical team will maintain the escalated dose for 24 h, and then follow hospital guidelines to evaluate subsequent changes in drug dose.
  - There are no limits on the number of escalations or resumptions of opioid for either intervention arm.
  - Escalation of the study drug dose is the mechanism to address NOWS signs that require additional pharmacotherapy per each hospital’s specific assessment tool. Spot doses or rescue doses are not part of this trial intervention
- The pharmacy will inform the clinical team when an infant has two dose levels remaining, which will allow the clinical team to be timely with discharge preparation.

### Post-hospital procedures

Primary caregiver(s) for infants for whom the protocol study team have obtained informed consent will receive questionnaires via electronic application or via phone interview, if caregiver(s) have limited access to cellular/internet service or prefer this modality of communication. Assessments may also take place in person, if there is a scheduled visit. Caregiver(s) will complete these questionnaires at 1 month after discharge and 6, 12, 18, and 24 months of age. These questionnaires will gather information on infant wellness, and primary caregiver(s) contact information, maternal well-being, infant attachment, and infant behavior. In addition, there will be an in-person follow-up visit with neurodevelopmental assessment at 24 months of age. Study staff will maintain contact in between study assessments at regular intervals. Respondents to these assessments will receive a reimbursement to compensate them for their time.

We will assess maternal well-being with Patient Reported Outcomes Measurement Information System (PROMIS) short forms [32]. Standardized short forms examining mental health, specifically the areas of anxiety (PROMIS Short Form v1.0 - Anxiety - 8a 31May2019), depression (PROMIS_SF_v1.0_-_ED-Depression_8a_5-31-2019), anger (PROMIS Short Form v1.1 - Anger - 5a 27Apr2016), life meaning and purpose (PROMIS Short Form v1.0 - Meaning and Purpose - 8a 18Jul2017), and social support (PROMIS v2.0 - Emotional Support Short Form 4a 23June2016), will be completed by the primary caregiver and will be sent to a central location for review by the protocol study team. The standardized short form for each of the PROMIS Measures consists of between four to eight 5-point Likert scale questions. The PROMIS Depression Short form has been validated in the postpartum period and has been found to be strongly correlated with the Edinburgh Postnatal Depression Scale, the most extensively studied measure of depression in the postpartum period [33, 34]. In addition, the PROMIS anxiety measure has been correlated with the Mood and Anxiety Questionnaire (MASQ) and has been shown to be a valid measurement tool for anxiety in the postpartum period in a sample of parents whose infants were hospitalized in the NICU [34]. Administration takes approximately 10 min and includes a total of 33 questions.

The PROMIS Measures will be administered at 1-month after discharge and again at 24 months of age.

We will briefly assess mother-infant attachment with the Maternal Postnatal Attachment Questionnaire (MPAQ; [35]), a 19-item questionnaire that assesses quality of bonding, absence of hostility, and pleasure in interaction. Higher MPAQ scores reflect higher levels of mother-infant bonding. The MPAQ requires approximately 5 min to complete, and researchers have validated the MPAQ among postpartum women with substance abuse problems [36]. The MPAQ will be administered at 1 month after discharge.

Caregiver(s) will complete the caregiver questionnaire (CQ) to assess enteral feeding, number of emergency room (ER) visits and/or acute/urgent care visits, and hospital readmissions.

We will assess infant neurobehavioral functioning at 24 months of age using the Brief Infant-Toddler Social and Emotional Assessment (BITSEA). The BITSEA is 42-item parent report screener used to indicate social-emotional/behavioral problems in children 12–36 months [37]. It will be administered at the 24-month in-person visit. We chose this measure because it is brief, easy to administer, and has good reliability and validity [38, 39].

We will assess infant development with the Bayley Scales of Infant and Toddler Development, Fourth Edition (Bayley-4): Cognitive, Language, Motor at 24 months of age. The Bayley-4 is recognized internationally as one of the most comprehensive tools to assess developmental outcomes in children. With the Bayley-4, it is possible to obtain detailed information even from non-verbal children as to their functioning. Children are assessed in the 3 key developmental domains of cognition, language, and motor. Reliability and validity of the previous version of the instrument have been well established [40].

### Potential risks and benefits to participants

#### Rapid wean

The rapid wean schedule is used routinely as standard of care at some U.S. hospitals. Among infants in the rapid-wean intervention arm, potential risks of the study intervention include a recurrence of NOWS signs and need to escalate and/or resume opioid treatment. If this trial is successful, potential benefits of the rapid-wean intervention include a shorter duration of opioid treatment, and possibly a shorter length of hospital stay.

#### Slow wean

The slow wean schedule is used routinely as standard of care at some U.S. hospitals. Among infants in the slow-wean intervention arm, potential risks include a longer duration of opioid treatment. Benefits of the slow-wean intervention include potentially fewer recurrences of NOWS signs.

#### Primary caregiver well-being

The research team will assess primary caregiver well-being (e.g., parenting stress, attachment, and bonding, depression, anxiety) during the follow-up portion of the study. Primary caregiver well-being will be assessed via the five PROMIS Measures and MPAQ questionnaires. It is possible that these questionnaires may reveal that the primary caregiver is experiencing psychological distress potentially requiring support. Mothers who have exposure to opioids during pregnancy may be vulnerable to suicidal ideation.

The study team has determined that a standardized scoring threshold for the PROMIS Depression Measure will be used to identify these individuals. As thresholds specific to postpartum women with opioid dependency have yet to be established and given that severe depression (a *t*-score >70, or 2 standard deviations above the mean for the normative population is the threshold for severe depressive symptoms) [41, 42] is most likely to impact family well-being, a score of >70 was chosen for this threshold.

If a primary caregiver has a *t*-score >70 on the PROMIS Depression measure, the primary caregiver will be provided with national hotline support numbers within the electronic questionnaire platform. In addition, after the questionnaire is completed in REDCap, an email will be automatically generated and sent to the study coordinator and PI. Each site will develop a plan to provide support for the primary caregivers at risk and connect them with local mental health resources in response to those emails. The protocol study team will collect a copy of this plan from each site.

We will train all personnel who administer the PROMIS Measures and MPAQ for appropriate responses if the caregiver expresses suicidal thoughts. This training will include additional questions to gauge the severity of the situation. We will require each hospital to develop a safety plan to provide the research team member immediate access to the Principal Investigator, designee, or other qualified individuals for further evaluation and direction. If there is an immediate concern by the research team member, knowledge of how to access local emergency responses will be available.

#### Maternal opioid use reporting requirements

The responsibility for determination of whether neonatal opioid exposure warrants mandatory reporting will rest with all mandatory reporters per requirements of those reporters. Participation in the clinical study will not affect reporting requirements.

### Criteria for discontinuing or modifying allocated interventions {11b}

#### Exiting the study intervention

Infants will exit the study intervention without unblinding (but remain in the trial) if they have not weaned off study drug by 35 days (inclusive of the 35th day) from the first weaning dose. This represents more than twice the median and mean length of treatment for the morphine arm in the Davis et al. trial [18]. This will avoid prolongation of treatment and length of hospital stay due to inability to tolerate the intervention guideline.

#### Other criteria to exit the intervention

- Participant who cannot ingest anything by mouth and needs intravenous opioid due to an increase in acuity or need of an operative procedure.
- Unable to take enteral opioid medication
- Participant who has a serious adverse event (SAE), including seizures, increased respiratory support, or intravenous fluid for increased stool output.
- Parents or legal guardians wish to withdraw their infant from the intervention.
- The clinical team feels it is in the best interest of the infant to be withdrawn from the intervention.

### Strategies to improve adherence to interventions {11c}

Strategies to improve or monitor adherence to the study protocol will include the following:

- Monthly recruitment reports of infants screened and enrolled (accrual figures)
- Monthly reports detailing data received at the NRN Data Coordinating Center (DCC), data consistency, missing data, performance measures, and adherence to the study protocol (with appropriate measures taken to preserve the blinding of study personnel and investigators)
- Supplementary blinded reports requested by the study investigators or subcommittee that do not disclose allocation group-specific outcomes (primary, secondary, or any safety outcomes).

### Relevant concomitant care permitted or prohibited during the trial {11d}

#### Control or monitoring of co-interventions

The clinical team may initiate treatment of NOWS signs with second- and third-line drugs after randomization, per hospital indications. The clinical team may escalate or wean the dose of these drugs during the study intervention per hospital guidelines.

### Provisions for post-trial care {30}

Similar to clinical practice, the clinical team should monitor participants who have weaned off study drug for 48–72 h prior to discharge to ensure that recurrence of NOWS signs do not occur. If there is a recurrence of NOWS signs during the 48–72 h post-intervention period, and if that recurrence merits pharmacologic therapy per the institution’s guideline, study drug will be restarted at the prior dose of the rapid wean (25% of stabilization dose) or the slow wean (20% of the stabilization dose) interventions. Tolerance for weaning will then be re-evaluated after 24 h of study drug administration. When the infant has been off the opioid and prior to discharge, a trained examiner will administer the NNNS. The assessment takes approximately 20–25 min to complete. We will not administer the NNNS at the same time relative to the last opioid exposure due to the unpredictable number and timing of opioid escalations in each weaning intervention and to avoid potential unblinding of the intervention.

### Outcomes {12}

#### ***Primary outcome***

The primary outcome will be the number of days of opioid treatment (used as primary treatment), including escalation, resumption, and spot treatment, from the first weaning dose to opioid cessation. We will assess the primary outcome by analyzing data from all infants undergoing rapid-wean, compared to slow-wean, with morphine or methadone. Predefined secondary analyses will examine the results for each opioid separately. We will define days on a 24-h basis, e.g., 18 h will represent 0.75 days. We will express days and dosages to the nearest hundredth, and we will round up at five. Days of opioid treatment is a single outcome that will be a function of (a) the weaning algorithm and (b) the extent of recurrence of NOWS signs. The use of hospital guidelines combined with study protocol guidelines will ensure that NOWS signs deemed clinically important result in appropriate treatment of the infant.

#### Secondary outcomes

##### Secondary efficacy outcomes

- *Secondary Outcome 1*: The numbers of days of opioid treatment from the first weaning dose to cessation of opioid with rapid- and slow-wean interventions among infants treated with morphine.
- *Secondary Outcome 2*: The numbers of days of opioid treatment from the first weaning dose to cessation of opioid with rapid- and slow-wean interventions among infants treated with methadone.
- *Secondary Outcome 3*: The proportions of infants in the rapid- and slow-wean intervention arms who have an escalation or resumption of opioid medication during weaning.
- *Secondary Outcome 4*: The total amounts of opioid from the first weaning dose to cessation of opioid among infants in the rapid- and slow-wean intervention arms.
- *Secondary Outcome 5*: The initiation and escalation of second- or third-line drugs to treat NOWS signs from the first weaning dose to cessation of opioid in the rapid- and slow-wean intervention arms.

##### Secondary safety outcome

- *Secondary Outcome 6*: The proportion of infants in each intervention arm with safety outcomes of seizures (clinical or electroencephalogram [EEG]), excessive stool output, respiratory disturbances, and feeding tolerance.

##### Other secondary outcomes

- *Secondary Outcome 7*: The proportion of infants in each intervention arm with an atypical neurobehavioral profile prior to discharge on the NICU Network Neurobehavioral Scale (NNNS).
- *Secondary Outcome 8*: The lengths of hospital stay for each intervention arm.
- *Secondary Outcome 9*: Assessments of maternal well-being and maternal-infant attachment in each intervention arm.
- *Secondary Outcome 10*: Assessments of growth in each intervention arm.
- *Secondary Outcome 11*: Assessment of infant wellness after discharge and until 24 months of age in each intervention arm.
- *Secondary Outcome 12*: Assessment of infant development to 24 months of age in each intervention arm.

### Participant timeline {13}

See Additional file 1 for schedule of activities.

### Sample size {14}

#### Sample size and power estimates

Eligible infants are those in the ISPCTN and NRN sites that clinical teams are pharmacologically treating for NOWS with an opioid as the primary drug treatment and have a gestational age ≥ 36 weeks. We used the most recent randomized trial comparing morphine to methadone to determine sample size and power estimates [18]. In that trial, researchers enrolled 116 pharmacologically treated NOWS infants from February 2014 until March 2017. The research team randomly allocated 58 infants to morphine treatment and 58 infants to methadone treatment. The standard deviation for the morphine arm was 6.9 days, while the standard deviation for the methadone arm was 8.0 days. We used these statistics to derive sample size estimates (Table 3). The total sample size estimates given in Table 3 assume that the clinical team will treat 70% of enrolled infants with morphine. This trial will enroll a total of 502 infants (251 infants to each of the rapid and slow wean interventions) irrespective of the proportion of infants treated with morphine or methadone.

**Table 3:** Sample size estimates

**Power**	**N per arm**	**Total *N***	**Morphine**^a^ ***N***	**Methadone**^a^ ***N***	**LOT-difference**	**SD**	**Alpha**
**0.9**	**251**	**502**	**352**	**150**	**2**	**6.9**	**0.05**
0.9	296	592	414	178	2	7.5	0.05
0.9	337	674	472	202	2	8.0	0.05
0.85	214	428	300	128	2	6.9	0.05
0.85	253	506	354	152	2	7.5	0.05
0.85	288	576	403	173	2	8.0	0.05
0.80	187	374	262	112	2	6.9	0.05
0.80	221	442	309	133	2	7.5	0.05
0.80	252	504	353	151	2	8.0	0.05

*LOT* Length of treatment days, *SD* Standard deviation

^a^Includes infants randomized to either rapid-wean or slow-wean interventions

A difference of 2.0 days in the length of treatment represents the minimum clinically important treatment effect for clinical care. If we can demonstrate a 2.0-day difference, there will likely be a reduction in hospital resources (bed, nursing, pharmacy, and physician) and cost. In addition, this effect size should facilitate a faster transition out of the hospital and keep the maternal-infant dyad together in a better environment than a hospital. The proposed intervention difference is similar to a recent trial comparing morphine to methadone [18]. Group sample sizes of 251 infants per treatment arm (total enrollment, 502 infants) will achieve 90% power to reject the null hypothesis of equal means when the population difference is 2.0 days with a standard deviation of 6.9 days and with a significance level of 0.05 using a two-sided two-sample *t*-test. If the standard deviation is as high as 8.0 days, a similar sample size will achieve 80% power to reject the null hypothesis using a two-sample *t*-test and a two-sided significance level of 0.05.

With respect to analysis of the Bayley-4 at 24-month follow-up, if the follow-up rate is at least 60%, then with a two=sided alpha of 0.05 we will be able to detect a difference in any composite score of 5.7 or greater with at least 80% power and will be able to detect a difference in any scaled score of 1.06 with at least 80% power.

#### Interval sample size reassessment

We defined length of treatment for the proposed trial as the average number of days of opioid treatment from the first weaning dose to cessation of opioid treatment. Due to a lack of available studies with published parameter estimates, we based the standard deviation used in the power calculations (6.9 to 8.0 days) on the number of days of opioid treatment for the *entire* interval of drug treatment, from initiation to cessation of morphine or methadone treatment [18]. We anticipate that a smaller standard deviation may be present in the proposed trial since we are only studying weaning. To address the concerns that the standard deviation may be lower than 6.9 days for our primary outcome, we will perform an interval sample size reassessment.

The interval sample size reassessment will occur after 25% of the enrolled infants are medically ready for discharge, which will be 126 infants, assuming full enrollment of 502 infants. This coincides with the first Data Monitoring and Safety Committee (DSMC) safety review. The NRN DCC will re-estimate the sample size and provide the DSMC with this report. To re-estimate the sample size, we will use the pooled variance estimate calculated across both intervention groups from blinded data observed in 126 study participants. This blinded look at the interim data used for sample size refinement will not require any alpha adjustment in the final primary outcome analysis.

#### Available population

In the fall of 2017, a ISPCTN and NRN site survey found that during a 1-year period, there were approximately 2700 infants exposed to opioids, of which, medical professionals pharmacologically treated approximately 43%. Among those treated pharmacologically, medical professionals treated 76% with morphine, 24% with methadone, and 4% with buprenorphine. These observations provide a starting to point to estimate the number of participants available for this trial. However, there is some uncertainty regarding how many NOWS infants would meet the inclusion criteria without meeting any exclusion criteria (Exclusion Criteria, Section 1). In addition, there may be changes in hospital practices within the ISPCTN and NRN given ongoing NOWS initiatives. Multiple clinical trials have not achieved enrollment of the projected sample size [15, 17, and 18], and we expect low consent rates in this population. Use of the Recruitment Plan was associated with a 35% consent rate at Women and Infants Hospital for the methadone vs morphine trial conducted by Davis et al. [18] in contrast to 26% among all participating centers. Table 4 provides an estimate of the number of infants enrolled per year based on estimates of available infants ranging from 1250 to 750 and consent rates ranging from 20 to 30%. Twenty hospitals in the NRN and ISPCTN have expressed interest in participation in this clinical trial.

**Table 4:** Estimated enrollment per year

**Consent rate**	**Enrollment by consent rate and infant numbers**		
	1250 available infants	1000 available infants	750 available infants
20%	250	200	150
25%	313	250	188
30%	375	300	225

### Recruitment {15}

There are 3 major components to a successful recruitment plan as follows:

1. Understand who is providing care for pregnant patients with an opioid use disorder.
2. Disseminate information to clinics, healthcare providers, and the medical community regarding research initiatives coupled with hospital care of the mother and newborn.
3. Identify pregnant mothers prior to delivery and use prenatal consultation to establish trust and provide an overview of newborn care and the clinical trial.

The first two of these components are part of a system-level recruitment initiative while the third component is patient specific. The single most important element of the recruitment strategy is the prenatal consultation. If prenatal consultation is not feasible, effective antenatal dissemination of information regarding the clinical trial will be exceptionally important when approaching mothers after delivery.

## Assignment of interventions: allocation

### Sequence generation {16a}

#### ***Randomization procedures***

##### Stratification

We will stratify randomization of infants by hospital. Stratifying by hospital will be critical to minimize the chance of differences between intervention arms in hospital practices, provider practices, and maternal characteristics. Stratification acknowledges that hospitals may have different practices than affiliated hospitals of a given center.

##### Randomization

We will randomly assign infants to intervention arms of either rapid weaning (15% decrements from the stabilization dose) or slow weaning (10% decrements from the stabilization dose). The Neonatal Research Network Data Coordinating Center (NRN DCC) will centrally randomize participants. They will develop an allocation sequence with randomly varying block sizes, and they will implement this sequence through a central process that will be available 24 h each day. The NRN DCC will independently randomize multiple births. Pharmacy personnel of each participating hospital will be the only staff with access to group assignment.

### Concealment mechanism {16b}

Because the randomization will occur through a central web-based application, site Principal Investigator (PI), research team, and clinical team will not be able to access the allocation sequence. To further add concealment, the act of randomizing is performed by the pharmacy team, which is the only unblinded service at each site. The act of randomization will be performed by the pharmacy team.

### Implementation {16c}

The NRN DCC will develop an allocation sequence with randomly varying block sizes, and they will implement this sequence through a central process that will be available 24 h each day. The NRN DCC will independently randomize multiple births. Pharmacy personnel of each participating hospital will be the only staff with access to group assignment.

## Assignment of interventions: blinding

### Who will be blinded {17a}

All site research personnel (site PI, research coordinator, research nurses), the clinical team (physicians, nurse practitioners, physician assistants, house-staff, medical students), family members, care givers legally responsible for the infant, hospital personnel (nurses, nursing assistants, clinical nurse managers, respiratory therapists, etc.), and any other personnel working in the area of the hospital where the infants is cared for will be blinded to the treatment intervention (rate of weaning). Only pharmacy personnel will not be blinded. All site research personnel responsible for administering questionnaires after discharge or performing evaluations at 2 years of age will be blinded. Participating hospitals will be responsible for in-services of all involved staff regarding blinding.

To maintain treatment blind, the volume of opioid will remain constant throughout the intervention for the rapid-wean and slow-wean intervention arms. At the time of opioid dosing, infants will receive one syringe with study drug at a volume equivalent to the volume of the stabilization dose, or a volume greater than the stabilization to facilitate maintaining a set volume (e.g., a stabilization dose of 0.28 ml may be set at 0.5 ml for ease of drawing up medication with saline during weaning). As infants progress through the dose levels, the research pharmacist will reduce the opioid volume and the pharmacist will make up the difference by normal saline so that the volume of the syringe is constant throughout all dose levels. To ensure that infants in the rapid-wean intervention arm have an equal number of study steps as infants in the slow-wean intervention arm, the pharmacy will use a placebo (normal saline without opioid) for three dose levels. Depending on the timing of escalations, the three placebo dose levels do not need to occur consecutively. We will label the study drugs as either morphine/study drug and the respective volume or methadone/study drug and the respective volume.

### Procedure for unblinding if needed {17b}

Infants will exit the study intervention without unblinding (but remain in the trial) if they have not weaned off study drug by 35 days from the first weaning dose. This represents more than twice the median and mean length of treatment for the morphine arm in the Davis et al. trial. This will avoid prolongation of treatment and length of hospital stay due to inability to tolerate the intervention guideline. To exit the Intervention one of the following criteria would need to be meet:

1. A participant cannot ingest anything by mouth and needs intravenous opioid due to an increase in acuity or need of an operative procedure.
2. Participant who has a serious adverse event, including seizures, increased respiratory support, intravenous fluid for increased stool output, or unable to take enteral medication.
3. Parents or legal guardians wish to withdraw their infant from the intervention.
4. The clinical team feels it is in the best interest of the infant to be withdrawn from the intervention.

If an infant meets one of the above criteria for exiting the intervention without unmasking, the following actions should be taken:

The clinical team responsible for the daily management of the infant will consult with the site research team. The site research team will verify that the circumstance for exiting the study intervention is appropriate by checking the Manual of Operations. Once verified, the site research team will communicate with the clinical team and the pharmacy that the infant will exit the study intervention. The pharmacy will provide the clinical team of what the opioid dose would have been in each arm of the trial.

### Sample Script: “If the infant was in the rapid wean arm they would have been receiving XX. If the infant was in the slow wean arm they would have been receiving YY.”

The clinical team will assume the management of the opioid medication using the clinical standards of the hospital. The treatment assignment will not be unmasked, and the infant continues as a study participant.

The actual dose that the infant is receiving will only be divulged to the clinical team if the physician caring for the infant feels that this information is essential for clinical management of the infant.

## Data collection and management

### Plans for assessment and collection of outcomes {18a}

Study Objectives and Endpoints

**Table:** 

**Objectives**	**Endpoints**
**Primary**	
• To evaluate the efficacy of a rapid-wean intervention compared with a slow-wean intervention in reducing the number of days of opioid treatment from the first dose of weaning to cessation of opioid among infants receiving an opioid (defined as morphine or methadone) as the primary treatment for neonatal opioid withdrawal syndrome (NOWS)	• The number of days of opioid treatment from the first dose of weaning to cessation of opioid
**Secondary**	
• To evaluate the efficacy of a rapid-wean intervention compared with a slow wean intervention in reducing the number of days of opioid treatment from the first dose of weaning to cessation of opioid among infants treated with morphine as the primary treatment for neonatal opioid withdrawal syndrome (NOWS)	• The number of days of morphine treatment from the first dose of weaning to cessation of morphine
• To evaluate the efficacy of a rapid-wean intervention compared with a slow wean intervention in reducing the number of days of opioid treatment from the first dose of weaning to cessation of opioid among infants treated with methadone as the primary treatment for neonatal opioid withdrawal syndrome (NOWS)	• The number of days of methadone treatment from the first dose of weaning to cessation of methadone
• To determine whether a rapid- or slow-wean intervention affects escalation or resumption of opioid medication during weaning among infants receiving an opioid (defined as morphine or methadone) as the primary treatment for neonatal opioid withdrawal syndrome (NOWS)	• Escalation or resumption of morphine or methadone medication during weaning
• To determine whether a rapid- or slow-wean intervention affects the total amounts of opioid given from the first dose of weaning to cessation of opioid among infants receiving an opioid (defined as morphine or methadone) as the primary treatment for neonatal opioid withdrawal syndrome (NOWS)	• The total amount of morphine or methadone given from the first dose of weaning to cessation of opioid
• To determine whether a rapid- or slow-wean intervention affects the administration of second- or third-line drugs to treat NOWS from the first dose of weaning to cessation of opioid among infants receiving an opioid (defined as morphine or methadone) as the primary treatment for neonatal opioid withdrawal syndrome (NOWS)	• Initiation or escalation of second- or third-line drugs administered to treat NOWS signs from the first dose of weaning to cessation of opioid
• To evaluate the safety and tolerability of a rapid-wean intervention compared with a slow-wean intervention among infants receiving an opioid (defined as morphine or methadone) as the primary treatment for neonatal opioid withdrawal syndrome (NOWS)	• Seizures (clinical or EEG), excessive stool output, respiratory disturbances, and feeding tolerance
• To determine whether a rapid- or slow-wean intervention affects neurobehavior among infants receiving an opioid (defined as morphine or methadone) as the primary treatment for neonatal opioid withdrawal syndrome (NOWS)	• Atypical neurobehavioral profile prior to discharge on the NICU Network Neurobehavioral Scale (NNNS) after completion of study drug and prior to hospital discharge
• To determine whether a rapid- or slow-wean intervention affects the total length of hospital stay among infants receiving an opioid (defined as morphine or methadone) as the primary treatment for neonatal opioid withdrawal syndrome (NOWS)	• The total number of days spent in the hospital
• To determine whether a rapid- or slow-wean intervention among infants receiving an opioid (defined as morphine or methadone) as the primary treatment for neonatal opioid withdrawal syndrome (NOWS) affects maternal well-being and maternal infant attachment at 4 weeks (± 7 days) after discharge	• Parent-Reported Outcome Measure Information System (PROMIS) Measures at 1 month after discharge and at 24 months of age • Maternal Post Attachment Questionnaire (MPAQ) at one month after discharge
• To determine whether a rapid- or slow-wean intervention among infants receiving an opioid (defined as morphine or methadone) as the primary treatment for neonatal opioid withdrawal syndrome (NOWS) affects growth over the first 24 months of age	• Weight (kg), length (cm), head circumference (cm), and weight for length percentile on the World Health Organization (WHO) growth curves. Anthropometric z-scores and Body mass index (BMI)-z at 24 months of age
• To determine whether a rapid- or slow-wean intervention among infants receiving an opioid (defined as morphine or methadone) as the primary treatment for neonatal opioid withdrawal syndrome (NOWS) affects infant wellness after discharge and until 24 months of age	• Acute/urgent care and/or ER visits (total number of occurrences) (CQ) • Readmissions (number of occurrences) (CQ) • Death (presence or absence)
• To determine whether a rapid- or slow-wean intervention among infants receiving an opioid (defined as morphine or methadone) as the primary treatment for neonatal opioid withdrawal syndrome (NOWS) affects infant development.	• Bayley Scales of Infant and Toddler Development, Fourth Edition (Bayley 4): Cognitive, Language, Motor, at 24 months of age

### Plans to promote participant retention and complete follow-up {18b}

To support retention of study participants after discharge, sites are instructed to use any retention resources at their institution including follow-up clinics and other similar resources. Sites should update contact information at every study follow-up timepoint. Participants will receive $50 compensation for each follow-up timepoint (1 month post-discharge, 6, 12, and 18 months) except 24 months, at which they will receive $100. Each site should use the appropriate type of participant reimbursement at their hospital. It is critical to keep participants informed and engaged during their participation in the study. Make sure that all of the contact timepoints are clearly communicated with each participant. After a participant enters the study, the site can follow up with a thank you note. Sites may also use text messaging to keep in contact with participants to update contact information and send reminders. Sites should check hospital records for updated contact information if they are unable to reach the participant by text, email, phone, or letter. Sites should document every attempt to contact a participant. More frequent contacts with the care giver (phone, text, email, etc.) in between study follow-up time points is encouraged but left to the discretion of the participating hospital. To maximize success rate in follow-up, it is recommended that participants are contacted/seen as early as possible in the following time windows:

- 1 month post-discharge: ± 3 weeks
- 6, 12 and 18 months: ± 6 weeks
- 24 months: 22–28 months. If an appointment was made for a 24-month evaluation prior to 28 months, the data will be used provided the evaluation is performed prior to 30 months.

Follow-up personnel/coordinators should anticipate that it will take multiple attempts to reach the caregiver. Attempts to contact a participant should be made on different days/times for a particular follow-up timepoint. If a family is unable to be contacted during one of the designated follow-up periods, they should be contacted again during the next follow-up period. If contact is made via telephone, and the family has limited time available for questionnaires, questionnaires should be administered in the following order:

- 1 month post-discharge: PROMIS, MPAQ, CQ
- 6 months: CQ
- 12 months: CQ
- 18 months: CQ
- 24 months: Bayley-4, BITSEA, PROMIS, CQ

Follow-up programs should anticipate that multiple attempts to establish contact will be necessary. If the site research team is having difficulty contacting a participant, the number of continued attempts should be discussed with the participating site/hospital PI. Regular meetings of the site/hospital research team and those involved in follow-up may be of benefit to devise strategies to maximize retention.

Critical to maintaining compliance for follow-up visits is the contact form collected during the recruitment period. The contact form incorporates information including the address and phone contacts of the parent/caregiver, as well as any other person listed as additional contacts. At the initial visit with the parent/caregiver and on all subsequent visits, contact information should be reviewed and updated. Two-way communication with the family needs to be implemented at each site through the parent’s preferred method of communication(s) including, telephone calls, text messages, emails, and face-to-face interactions. Seasonal/birthday cards, mailings, and/or newsletters are encouraged because it keeps participants engaged, as well as provides address verification. Returned mailings should be updated in the tracking system. The use of private messages, similar to email, may be allowed and an effective tracking tool when other contact information is no longer in use.

Before hospital discharge, the study team should obtain information from the mother or other caregivers so that other relatives or friends could be contacted to find the family’s new address if they had moved. It is important that families and caregivers be made aware that any information regarding address or phone number remain strictly confidential and will not be shared with anyone for any other purpose but making appointments for the infants’ benefit.

### Data management {19}

#### Data management

RTI International will provide the following:

- Collaborates in the development, implementation, and monitoring of Weaning protocol.
- Provides biostatistical leadership in statistical design aspects of Weaning protocol.
- Provides data management, including development of case report forms (CRFs) and appropriate data collection systems.
- Supervising data entry activities, including instructing and certifying data entry personnel in software and hardware usage, and quality assurance of data entry.
- Designs and maintains central randomization system.
- Manages the Data Safety and Monitoring Committee for the trial, including scheduling meetings, the DSMC charter and preparing interim monitoring reports for the DSMC.
- Oversees the receipt and reconciliation of safety data.
- Supervises NRN site quality assurance efforts, including conducting site visits and remote monitoring of data.
- Prepares and distributes monthly reports, detailing data received, data consistency, miss data and adherence to protocol.
- Disburses capitation payments to clinical centers on the basis of enrolled patients and other study-specific milestone triggers specified in the study protocols.
- Provides the logistical support necessary to run an efficient and productive network.
- Provides biostatistical leadership for collaborative analysis of study data and publication of results.
- Prepares public-use data files.

### Confidentiality {27}

Study identifiers will be strictly protected in this study. Subjects are assigned a unique study identification number, which is used on all electronic data files and study forms with the exception of the screening log. Identifying information such as name, date/time of birth, and medical record number (MRN) will be used on screening log for documentation and tracking purposes. Name and MRN will not be entered into the online study databases RAVE and REDCap. The password-protected screening log will be securely stored on hospital password-protected computers only. The log will be destroyed at the end of the study. Confidentiality will be protected by removing any information that can be associated with date/time of birth and other dates/time data points. The link between the identifying numbers and names will be securely locked and accessible only to personnel of the Weaning Trial including study coordinator and PI. Data for the in-hospital weaning data collection forms will be entered into the Medidata RAVE Electronic Data Capture (EDC) system. Medidata RAVE is a CFR 21 Part 11 compliant Electronic Data Capture System with ISO 27018 privacy protection certification. The RAVE system is an interface for data capture, queries, data cleaning, and site monitoring and reporting.

In-hospital weaning data abstraction and collection forms will be entered into the web-based online EDC system RAVE database electronically, verified and stored without identifying information. REDCap EDC will be used for the post-hospital discharge questionnaires and follow-up data. Participants can complete study questionnaire electronically via REDCAP. These electronic data files are linked to the participant by the unique participant identification number only.

### Plans for collection, laboratory evaluation, and storage of biological specimens for genetic or molecular analysis in this trial/future use {33}

Not applicable; we will not collect biological specimens during the conduct of this study.

## Statistical methods

### Statistical methods for primary and secondary outcomes {20a}

#### Analysis of the primary hypothesis and outcome

We will determine the outcome of the primary hypothesis on an intention to treat basis. We will assign infants who exit the intervention at 35 days of methadone/morphine treatment either as 35 days of opioid treatment or we will treat them as a censored value at 35 days. We will determine intervention differences of two means by analyzing the average number of days of opioid treatment from the first weaning dose to cessation of opioid treatment. We will analyze the data using regression models that will include the intervention as a fixed effect and will include maternal treatment and stabilization dose as covariates. We will include site (hospital) in the model as a random effect. The primary test of interest will be the F-test of the intervention effect, and we will report the intervention difference along with 95% confidence interval (CI).

Because of possible censoring and removal of participants due to intervention failure, we will conduct sensitivity analysis of the primary outcome using several methods. The first sensitivity analysis will replicate the analysis, described above, and include other covariates in the model that were significantly different across the two weaning intervention groups. Sensitivity analyses will also be conducted to evaluate the effects of infants who were and were not part of the Eat-Sleep Console trial as a covariate or effect modifier. Another possible sensitivity analysis will include non-parametric and/or survival regression (e.g., negative binomial, median regression, or survival analyses), as there is potential for skewness and censoring in the primary outcome.

Finally, sensitivity analysis will include fitting a competing risks model to the data where the possible competing risk states are weaned, parental withdrawal, physician withdrawal, and treatment failure (unable to wean by 35 days of methadone/morphine treatment). We will fit Cox proportional hazards models to the data to estimate the intervention effect and other covariate effects on “cause-specific hazards.” The analysis of the “cause-specific hazards” will allow for additional inquiry into the intervention effect on the primary outcome while accounting for competing safety and withdrawal risks.

Descriptive statistics (means, medians, SD, percentiles) for number of days of opioid treatment from the first weaning dose to cessation of opioid treatment will be generated and summarized in a table by treatment group.

#### Analyses of secondary outcomes

We will use the same approach described in the primary outcome analysis to compare the number of days of opioid treatment with only infants receiving morphine as the primary pharmacological treatment for NOWS (Secondary Outcome 1). We will use this same approach to compare the number of days of opioid treatment using only infants receiving methadone as the primary pharmacological treatment for NOWS (Secondary Outcome 2).

We will use adjusted logistic regression models to provide an odds ratio (OR) and 95% confidence intervals (CI) for binary secondary outcomes measured only once. These outcomes will include:

- The proportion of infants by intervention arms that escalate or resume opioid medication during weaning,
- For the proportion of infants by intervention arms with an atypical neurobehavioral profile,
- The proportion of infants who receive an escalation of a second-line or third-line drug to treat NOWS signs from the first weaning dose to cessation of opioid.

We will adjust these models for the stratification variable (hospital), covariates of maternal treatment and stabilization dose, and baseline variables that may differ between groups by chance. We define the covariates that we may adjust for earlier in this section in the paragraph on other collected data.

We will analyze the secondary outcome of the total opioid exposure from the first weaning dose to cessation of opioid in a similar manner as the primary outcome. We will use a regression model that will include a fixed treatment effect (intervention arms) and adjustment for the stratifying variable, covariates of maternal treatment and stabilization dose, and baseline variables that may differ between groups by chance as fixed effects. We will include hospital (site) as a random effect. The primary test of interest will be the F-test of the intervention arm effect, and we will report the treatment difference along with 95% CI.

We will measure the secondary outcome of maternal well-being and maternal-infant attachment by using PROMIS Measures and MPAQ total scores. We will analyze these in a similar manner as the primary outcome. We will use a regression model to analyze PROMIS Measures and MPAQ total scores. This model will include a fixed treatment effect (intervention arms), an adjustment for the stratifying variable, and fixed effects for the covariates of maternal treatment and stabilization dose. The PROMIS Measures outcomes are measured at 1-month after discharge and 24-months of age. The models for PROMIS Measures outcomes will include the 1-month after discharge PROMIS Measures outcome as a covariate. The F-test of the intervention arm effect will be the primary test of interest. We will report the intervention arm difference along with 95% CI. We will conduct analyses among the entire group and among those where the biologic mother is the primary caretaker.

We will measure the secondary outcome of infant neurobehavioral functioning using BITSEA scores. The BITSEA consists of two multi-item scales, a Problem scale (31 items) and a Competence scale (11 items). A high score on the Problem scale or a low score on the Competence scale is less favorable. We will analyze these in a similar manner as the primary outcome. We will use regression models to analyze the BITSEA total and subscale scores. This model will include a fixed treatment effect (intervention arms), an adjustment for the stratifying variable, and fixed effects for the covariates of maternal treatment and stabilization dose. The F-test of the intervention arm effect will be the primary test of interest. We will report the intervention arm difference along with 95% CI. We will conduct analyses among the entire group and among those where the biologic mother is the primary caretaker.

We will calculate binomial proportion and their corresponding 95% CI by intervention arm for each of the following safety adverse events: seizures (clinical or EEG), excessive stool output, respiratory disturbances, and feeding tolerance. We will use chi-square tests to compare the proportion of seizures (clinical or EEG), excessive stool output, respiratory disturbances, and feeding tolerances between intervention arms. In addition to unadjusted analyses, we will compare AEs across the weaning interventions by using adjusted logistic regression models that adjust for the stratification variable (hospital), covariates of maternal treatment and stabilization dose, and possibly any baseline variables that were significantly different between weaning interventions. We define covariates that we may adjust for earlier in this section within the paragraph on other collected data.

We will analyze the length of hospital stay in a similar manner as the primary outcome. We will use a simple, unadjusted regression model to analyze the total length of stay and will include a fixed treatment effect (intervention arms). A second regression model will include a fixed treatment effect and will include adjustment for the stratifying variable and covariates of maternal treatment and stabilization dose. The F-test of the treatment effect will be the primary test of interest. We will report the treatment difference along with 95% CI. Sensitivity analysis will involve time-to-event analyses using survival models to account for skewness and possible censoring of each outcome.

We will analyze the caregiver questionnaire outcomes and the death outcome using a longitudinal generalized linear mixed-effects model (GLMM) or generalized estimating equations (GEE) model appropriate for the outcome type since the data will be collected at multiple time points after discharge. Count data that tend to have more than 0 or 1 events counted will be analyzed using a Poisson model while binary or count data that rarely goes beyond 1 occurrence will be analyzed using a Logistic model. In case of count data that rarely goes beyond 1 occurrence, this data will be transformed to binary data (occurrence/no occurrence). We will present mean outcome ratios for count data (from Poisson models) and odds ratios for binary data (from logistic models) with respect to the intervention effect as well as 95% CI of the intervention effect. All analyses will be adjusted for repeated measures over time, so that patterns of change for these outcomes over time can be assessed by treatment group.

In addition to time of discharge and 24 months of age, anthropometric outcomes will be measured at birth. We will calculate anthropometric *z*-scores at each of the three assessment periods for the purpose of analysis based on age and gender-specific World Health Organization norms. The approach to analyzing weight is given next.

We will provide the mean and SD of infants’ weights (*z*-scores) separately for each treatment group. We will use a mixed linear model to evaluate the effect of treatment arm on weight (*z*-scores). The model will examine how the treatment means differ (i.e., main treatment effect), how treatment means change over time (i.e., main time effect), and how differences between treatment means change over time (i.e., treatment-by-time effect). We will carry out assessment across 3 time points: birth, hospital discharge, and 24 months of age. The mixed model longitudinal analytical approach allows us to analyze correlated data obtained repeatedly from the same participant and account for the intraclass correlation among participants nested within with same clinical site. To account for potential imbalance in key demographic and site-level characteristics, unadjusted and adjusted GLMMs will be fit to the data. Initially, the unadjusted mixed model will include the fixed categorical effects of intervention, time, and intervention-by-time interaction and the random-site effect. We will calculate the point estimates and their respective CIs for the changes in infants’ weights for each intervention group and for the difference in the estimated change between intervention groups. Additionally, the team will present the *p*-value of the difference in point estimates between intervention groups.

We will examine the impact of the treatment arm on length, head circumference (HC), and infant weight for length (*z*-scores) using the same analytical methods described for weight (*z*-scores) above. Additionally, we will provide the mean and SD of infant BMI-z at 24 months for each treatment group. The team will use a GLMM with an identity link to compare average BMI-z between the groups, and the team will report point estimates for the group mean difference along with a 95% CI.

To compare Bayley-4 scores between intervention arms, we will perform a linear mixed-effects model with a fixed effect for the intervention group and a random effect for study site. We will report point estimates for the group mean difference along with a 95% CI, and the team will repeat this analytical approach for each of the Bayley-4 domains.

Descriptive statistics (means, medians, SD, percentiles) for continuous secondary outcomes and frequency-based statistics (N and percentages) for binary secondary outcomes will be generated and summarized in a tabular form by treatment group.

### Interim analyses {21b}

#### ***Interval sample size reassessment***

We defined length of treatment for the proposed trial as the average number of days of opioid treatment from the first weaning dose to cessation of opioid treatment. Due to a lack of available studies with published parameter estimates, we based the standard deviation used in the power calculations (6.9 to 8.0 days) on the number of days of opioid treatment for the *entire* interval of drug treatment, from initiation to cessation of morphine or methadone treatment [18]. We anticipate that a smaller standard deviation may be present in the proposed trial since we are only studying weaning. To address the concerns that the standard deviation may be lower than 6.9 days for our primary outcome, we will perform an interval sample size reassessment.

The interval sample size reassessment will occur after 25% of the enrolled infants are medically ready for discharge, which will be 126 infants, assuming full enrollment of 502 infants. This coincides with the first Data Monitoring and Safety Committee (DSMC) safety review. The NRN DCC will re-estimate the sample size and provide the DSMC with this report. To re-estimate the sample size, we will use the pooled variance estimate calculated across both intervention groups from blinded data observed in 126 study participants. This blinded look at the interim data used for sample size refinement will not require any alpha adjustment in the final primary outcome analysis.

#### Data monitoring plan and stopping rules

There is wide variability in weaning opioid drug treatment across IDeA States and NRN hospitals. Well-characterized AEs and a DSMC will be critical to monitor the trial and to assure that the interventions are safe. The DSMC will assess safety after 25, 50, and 75% of the enrolled infants are medically ready for discharge, and it will assess efficacy and futility after 50% of the enrolled infants are medically ready for discharge. All interim analyses will utilize Bayesian modeling and predictive posterior inference based on neutral, enthusiastic, and skeptical priors. We will use Bayesian modeling for the interim analyses because the predictive posterior inference makes a clear statement about what to expect when we complete frequentist analysis on final data, given the interim results. The DSMC will receive an independent presentation of interim results, prepared by the study statistician. In preparation for the DSMC meeting, we will prepare a summary report of recruitment (by hospital), known outcome events, and any AEs (including medication side effects).

##### Interim futility and efficacy analysis

For interim efficacy, we will use Bayesian posterior predictive probabilities to predict the final outcome of the trial based on interim results. For this predictive probability calculation, we will use a frequentist criterion: reject null hypothesis if final analysis *p*-value is less than or equal to 0.05. Given this criterion and the neutral, enthusiastic, and skeptical priors defined above, we will calculate three predictive probabilities of success (PPoS) when 50% of the total sample is collected by using the three reference priors: neutral, skeptical, and enthusiastic. To calculate a PPoS, we will take the following steps:

(4) Choose the neutral, enthusiastic, or skeptical prior for the treatment effect.
(5) Fit a Bayesian linear regression model to the primary outcome using the interim data. Include maternal treatment and stabilization dose as covariates.
(6) Calculate the posterior distributions for all regression terms.
(7) Use the interim data to calculate the observed distribution of maternal treatment.
(8) Use the interim data to calculate the observed distribution of the stabilization dose. May approximate with a normal distribution if suitable.
(9) Determine how many infants we still need to randomize in each arm.
(10)  For each arm, generate data for the required number of hypothetical participants by doing the following for each required hypothetical participants:
  Sample a single value from each posterior distribution.
  Make a random draw from the observed distribution for maternal treatment.
  Make a random draw from the observed or approximated distribution for stabilization dose.
  Use the appropriate sampled values from steps 7a–7c to generate a hypothetical outcome.
(11)  Use the data observed plus the hypothetical data generated in Step 7, above, to create a hypothetical complete trial and calculate the *p*-value under the null hypothesis of *θ* = 0. Use a linear regression model that includes treatment, maternal treatment, and stabilization dose.
(12)  Repeat Steps 7 and 8 many times. The PPoS is the proportion of hypothetical completed trials that achieve a *p*-value for the treatment effect that is 0.05 or less. The PPoS will be a helpful measure for the DSMC to use as it makes decisions about stopping the trial early for efficacy or futility or continuing to enroll. Below are two suggested guidelines for using the PPoS, but as stated in the DSMC charter, all protocol suggested stopping guidelines are advisory and the DSMC can choose to ignore them. If the PPoS is 0.99 or greater under the skeptical prior, then the DSMC may consider stopping the trial for efficacy. If the frequentist PPoS is 0.1 or less under the enthusiastic prior, then the DSMC may consider stopping the trial for futility. The PPoS under the neutral prior will also be available to aid interpretation of the PPoS estimates calculated under the skeptical and enthusiastic priors.

##### Interim safety analysis

In addition to monitoring AEs and SAEs, the DSMC will use Bayesian analyses to monitor seizure occurrence. The DSMC will assess seizure occurrence at three interim reviews, 25, 50, and 75% of enrollment. Within the slow-wean intervention, we expect the seizure proportion to be 0.03, and within the rapid-wean intervention, we expect this proportion to be higher. Based on clinical experience, the study team offers the following guideline for stopping the trial early for safety. Yet as with Efficacy and Futility, per the DSMC charter, all protocol suggested stopping guidelines are advisory and the DSMC can choose to ignore them. If the seizure proportion is 0.03 in the slow-wean intervention, and the seizure proportion is greater than 0.10 in the rapid-wean intervention, then the DSMC may consider stopping the trial for safety. As such, the interim analyses of safety will focus on reporting information about the seizure proportion in the slow-wean intervention and the difference between seizure proportions in both interventions. At each interim analysis of safety, we will calculate the posterior distribution of seizure proportion within each intervention by using a simple Bayesian logistic regression model, intercept and treatment effect parameters with neutral priors on the intercept and the intervention effect parameter. We will place a normal (*μ*=0, *σ*=10) on the intercept, and we will place a normal (*μ*=0, *σ*=3) on the intervention effect term. Let *θ*_1_ = proportion of infants with seizures in the rapid-wean intervention and *θ*_2_ = proportion of infants with seizures in the rapid-wean intervention minus the proportion of infants with seizures in the slow-wean intervention. We will calculate the posterior distribution of *θ*_1_ and *θ*_2_ by using transformations of the MCMC values that make up the estimated posterior distributions for the intercept and the intervention effect parameter. If Pr(*θ*_1_ > 0.1 | Data) ≥ 0.95, and Pr(*θ*_2_ > 0.07 | Data) ≥ 0.95, then the DSMC may want to consider stopping the trial for safety.

Under this guideline, the DSMC may consider stopping the trial for safety, if there is convincing evidence that the seizure proportion among rapid-wean infants is greater than 0.1 and there is convincing evidence that the seizure proportion among rapid-wean infants is more than seven percentage points greater than the seizure proportion among slow-wean infants. Finally, as with efficacy and futility, the DSMC charter states trial stoppage guidelines can be ignored if the DSMC determines it is necessary.

### Methods for additional analyses (e.g., subgroup analyses) {20b}

#### Bayesian analyses

Other randomized trials of NOWS have ended prior to meeting the projected enrollment, indicating the challenges in studying this population [15, 17, 18]. In case of insufficient enrollment, we will pre-specify Bayesian analyses of the final data in addition to the frequentist analyses, defined above. Below, we first define the Bayesian analyses that will mirror the above defined frequentist analyses of the primary outcome.

We will analyze the primary outcome with a linear regression that will include treatment group (intervention arms), stabilization dose, and maternal treatment as covariates, and we will include hospital as a random effect. We will use a neutral prior for treatment effect that is centered at a mean difference of 0 and a standard deviation of three, normal (*μ*=0, *σ*=3) distribution. For the intercept term, we will use a normal (*μ*=0, *σ*=10) prior. For all other baseline covariates in the model, we will use weakly informative neutral normal (*μ*=0, *σ*=2) priors. We will use a weakly informative half-normal (*μ*=0, *σ*=1) prior for the standard deviation of the random hospital effect. For all models, we will report posterior medians and 95% credible intervals (CrI) for the group mean differences and the probability that a rapid-wean intervention will reduce the days of opioid treatment, compared to a slow-wean intervention. For sensitivity analyses, we will analyze the primary outcome with skeptical and enthusiastic priors. We will center the skeptical prior at a mean difference of two, indicating the view of a skeptic with belief that the study intervention will increase the number of days of opioid treatment by 2 days. We will center the enthusiastic prior at a mean difference of −2, meaning a priori that an enthusiast expects the study intervention to reduce the number of days of opioid treatment by 2 days. In both the enthusiastic and skeptic priors, the standard deviation will be three. As with the primary frequentist analysis, sensitivity analyses may be done using Bayesian analyses, where normal (*μ*=0, *σ*=2) priors will be placed on all covariates.

##### Subgroup analysis for secondary outcomes 1 and 2

To estimate possible treatment effect heterogeneity for the primary outcome, we will use a Bayesian hierarchical model with main effects and interaction term between intervention group and type of narcotic (morphine or methadone). This approach allows us to specify a priori how likely (or unlikely) it is for subgroup differences to be present and to shrink the subgroup estimates to the overall mean treatment effect. We will specify a model that allows different standard deviations of the outcome for the two subgroups (as observed in Davis et al.). Prior distributions for main effects will be the same as for the primary outcome. Neutral and skeptical priors will be used for the interaction terms. Point estimates of treatment effect and 95% credible intervals will be reported for each subgroup along with probability of subgroup treatment differences.

We will analyze secondary binary outcomes with Bayesian logistic regression models, including treatment group, maternal treatment, and stratification dose as covariates, and we will include hospital as a random effect. We will use a neutral prior centered at 1.0 with 95% CrI of 0.33–3.0 (to allow for large range of ORs) for the treatment effect. In the log OR scale, this prior will be normal (0, SD=0.57), and all other priors will be the same as above.

We will analyze secondary outcomes of the total opioid exposure and assessments of maternal well-being, maternal-infant attachment, infant neurobehavioral functioning, infant development, and growth with a similar linear regression model used for the primary outcome.

We will analyze secondary outcome of length of stay with a linear regression model similar to the one used to analyze the primary outcome; however, we will also fit survival analysis models to study the effects of skewness.

We will implement all Bayesian models via Markov chain Monte Carlo methods (MCMC) by using R or SAS software. For SAS, the procedure will be PROC MCMC. For R, possible software is “RJAGS” which is an interface to JAGS MCMC software, “rstan,” “rstanarm,” and “brms,” which are packages that interface with the Stan language. For each analysis, we will run three MCMC chains with randomly drawn starting values. We will use a burn-in of 3000 iterations, with sampling from a further 30,000 iterations for each chain. Thinning will be used as necessary to reduce autocorrelation among the samples to improve posterior sampling. To monitor convergence, we will use trace plots and the Gelman-Rubin convergence diagnostic (Rhat < 1.1 indicating convergence) for all parameters.

### Methods in analysis to handle protocol non-adherence and any statistical methods to handle missing data {20c}

Analysis will be completed on the intention to treat principle, so data will be analyzed as randomized. No imputation for missing outcome data is planned, so the analysis will be on the Full Analysis Set [43].

### Plans to give access to the full protocol, participant-level data, and statistical code {31c}

This study will comply with the NIH Public Access Policy, which ensures that the public has access to the published results of NIH-funded research. The study will also comply with the NIH Data Sharing Policy and Policy on the Dissemination of NIH-Funded Clinical Trial Information and the Clinical Trials Registration and Results Information Submission rule.

As such, this study will:

- Register with ClinicalTrials.gov and submit results. We will submit primary outcome results from this trial to ClinicalTrials.gov.
- Publish results. We will make every attempt to publish results in peer-reviewed journals. We will submit all final peer-reviewed journal manuscripts from this study to the digital archive PubMed Central upon acceptance for publication.
- Deposit data for data sharing with other researchers. Within the bounds of relevant IRB approvals and guidelines for protection of personally identifiable data, we will deposit de-identified data from this study in an appropriate, NIH-approved data repository.

## Oversight and monitoring

### Composition of the coordinating center and trial steering committee {5d}

NIH Program Officers at NICHD and ISPCTN-ECHO (in the NIH the Office of the Director (OD)) will be responsible for:

• Providing oversight for the trial.

• Facilitating and participating in all study-related committees.

• Funding the study, administering the Protocol Review Committee (PRC) which meets at a minimum twice a month.

• Administering the Data Safety and Monitoring Committee (DSMC) which meets on a quarterly basis.

• The NIH Program Officers at each of these institutions will have final authority on the commencement and continuation of the protocols.

Three coordinating centers will collaborate to perform all coordination activities for the ACT NOW collaborative:

1. ECHO Coordinating Center (CC) – Duke Clinical Research Institute (DCRI)
2. NRN Data Coordinating Center (DCC) – RTI International
3. ECHO ISPCTN Data Coordinating and Operations Center (DCOC) – University of Arkansas for Medical Sciences (UAMS)

The three coordinating centers will be jointly responsible for working with the Clinical Sites on study implementation. Activities include training, data management and biostatistical management. Responsibilities for each organization are outlined in the subsections below.

The CC, DCRI, will:

- Be responsible for the daily operations across all non-NRN sites (e.g., ISPCTN sites and other sites)
- Monitor the performance of all non-NRN clinical sites and meet with NICHD and NIH on a regular basis to review site performance.
- Administer site contracts with non-NRN sites.
- Pay site capitation to Non-NRN sites.
- The DCC, RTI International, will:
- Be responsible for the daily operations across all NRN and several non-NRN sites.
- Collaborate in the development, implementation, and monitoring of Weaning protocol.
- Provide data management, including development of Case Report Forms (CRFs) and appropriate data collection systems
- Supervise data entry activities, including instructing and certifying data entry personnel in software and hardware usage, quality assurance of data entry, etc.
- Manage the Data Safety and Monitoring Committee (DSMC) for the trial. This will include scheduling meetings and the DSMC charter.
- Oversee the receipt and reconciliation of safety data.
- Supervise Neonatal Research Network (NRN)-site quality assurance efforts, including conducting site visits and remote monitoring of data.
- Prepare and distribute monthly reports, detailing data received, data consistency, missing data, and adherence to protocol.
- Disburse capitation payments to clinical centers on the basis of enrolled participants and other study-specific milestone triggers specified in the study protocol.
- Provide the logistical support necessary to run an efficient and productive network.
- The DCOC, UAMS, will:
- Act as the central IRB regulatory oversite for this protocol.

### Composition of the data monitoring committee, its role and reporting structure {21a}

The independent DSMC will have overall responsibility for interim data monitoring and operate based on the Institutional Development Award (IDeA) States Pediatric Clinical Trials Network (ISPCTN) and NRN charter for the DSMC. The DSMC meets regularly to review the ongoing ACT NOW Weaning protocol with respect to ethical and safety standards. It monitors the safety of the clinical trials and advises the on continued study conduct. The DSMC provides recommendations to the Directors of NICHD and ECHO about starting, continuing, and stopping the ACT NOW Weaning clinical trial. All data distributed to the DSMC and deliberations of the DSMC are strictly confidential. Decisions to alter or halt studies are the responsibility of the Directors of NICHD and ECHO. The DSMC may recommend protocol modifications based on concerns for patient welfare or scientific integrity. The committee has confidential access to statistical data and adverse events that it may require for its deliberations. It reviews interim reports of patient accrual and outcome measures provided by the Data Coordinating Center.

### Adverse event reporting and harms {22}

#### Adverse events

All study personnel will assess for adverse events (AE) from the start of study drug to hospital discharge (i.e., “study period” in corresponding CRF) while being blinded to the weaning intervention. Adverse events will include the following:

- *Seizures*: The clinical team will evaluate abnormal movements for potential seizure activity. A seizure is defined clinically as a paroxysmal change in neurological function including motor, behavioral, and/or autonomic function. If there is a high index of suspicion for seizures that results in a change of clinical management (e.g., escalation of care, initiation of anti-epileptic drugs, re-initiation or escalation of morphine/methadone for presumed seizure activity), infants should exit the weaning intervention and clinical management of NOWS should be assumed by the clinical team. We do not know the frequency of seizures during the weaning phase with current maternal and infant treatment. Researchers reported clinical seizures in 2 to 11% during the acute phase of abstinence from infants born in the 1960s to 1970s when treatment approaches differed [44–46]. EEGs were performed in a small group of infants in these reports (< 10%, [45, 46]), and firm conclusions cannot be drawn. More recently, small cohorts of infants at risk or treated for NOWS have been investigated with EEG. Amplitude integrated EEG recordings in 9 infants did not indicate seizures but did have abnormalities of background and sleep cycles [47, 48]. Among 40 infants with NOWS referred for clinical seizures, EEG, and video EEG indicated an abnormal background in 27.5% and electrographic seizures in 7.5% [48]. The latter does not represent the frequency of seizures among infants with NOWS since this report only reported on infants with presumed clinical seizures.
- *Stool output*: An increase in stool output that the clinical team treats with intravenous therapy.
- *Respiratory disturbances*: Tachypnea (respiratory rates > 80 beats per minute (bpm) consistently recorded over 4–6 h with decreases in oxygen saturation < 85%), shallow breathing (respiratory rates < 30 bpm consistently recorded over 4–6 h with decreases in oxygen saturation < 85%), or increased respiratory support (nasal cannula or greater for infants previously on room air).
- *Feeding strategy*: A change in feeding strategy (e.g., Intravenous [IV] fluids) due to poor feeding or emesis.
- *Other adverse events*: This will include any change in clinical status during the weaning interventions that is clinically significant by the Site Principal Investigator.

#### Serious adverse events

All study personnel will consider adverse events serious if they include any of the following:

1. Death
2. Life-threatening adverse event
3. Inpatient hospitalization or prolongation of existing hospitalization
4. Persistent or significant incapacity or substantial disruption of the ability to conduct normal life functions
5. Important medical events that may not result in death, be life-threatening, or require hospitalization, but based on medical judgment may jeopardize the participant and may require medical or surgical intervention to prevent one of the outcomes listed above in this definition.

Participants with serious adverse events will exit the intervention without unblinding the treatment intervention assignment, unless the clinical team considers unblinding essential to the provision of clinical care. The clinical team will assume the care of a participant who exits the intervention, and we will provide the current dose of opioid to the clinical team.

### Frequency and plans for auditing trial conduct {23}

#### ***Compliance monitoring***

Strategies to improve or monitor adherence to the study protocol will include the following:

- Monthly recruitment reports of infants screened and enrolled (accrual figures)
- Monthly reports detailing data received at the NRN DCC, data consistency, missing data, performance measures, and adherence to the study protocol (with appropriate measures taken to preserve the blinding of study personnel and investigators)
- Supplementary blinded reports requested by the study investigators or subcommittee that do not disclose allocation group-specific outcomes (primary, secondary, or any safety outcomes).

The DCC will generate the aforementioned reports.

#### Data quality monitoring and assurance

To assure the quality of the data collected, the trial investigators will conduct training sessions on protocol implementation, data acquisition, and data transfer. Sites will be required to attend a mandatory training session that engages multiple research team members, including at least one site investigator, one study coordinator, and one data entry staff. This training will consist of a walkthrough of the protocol, randomization procedures, study intervention, data collection procedures, the Manual of Operations (MOP), and demonstration of the electronic data capture system. The training will provide guidance specific to accuracy of data acquisition for the research coordinators at each site. The data collection forms will be piloted by a subset of sites to minimize the potential for errors. Additionally, in-depth pharmacy training will be held with site pharmacists that will consist of a walkthrough of the protocol, randomization procedures, study drug dispensing, blinding, and demonstration of the EDC. Sites will be required to attend both the protocol and pharmacy training prior to study launch. Sites will also be required to attend annual protocol refresher sessions until enrollment is complete.

The NRN DCC will employ a mixed method data quality monitoring approach that will involve a combination of the following methods: centralized monitoring, chart re-abstraction, and onsite monitoring.

#### Central monitoring

Central/remote monitoring will incorporate a variety of methods to detect and resolve potential data quality issues. Within the EDC, preprogrammed data edit checks (e.g., out-of-range values, required fields, skip patterns) will trigger queries to hospitals in real time (e.g., upon data entry). The NRN DCC will also manually review the data monthly, which may result in the data manager manually entering queries in the EDC that site study staff must complete. Email communications with the site will be used to resolve more complex questions about the data.

The NRN DCC will generate study-level and hospital-level status reports that will be updated and reviewed on a monthly basis. These reports will identify issues such as missing forms, major protocol violations, or safety events that require follow-up. The trial subcommittee will then discuss these study-level and hospital-level status reports on monthly subcommittee calls to identify overall study and hospital trends that suggest deviations from the specified protocol procedures, data quality concerns, or occurrence of safety events of concern. Sites identified with concerning trends will meet with selected members of the trial subcommittee to discuss the errors and potential solutions. Following the conference call, if the site is identified again with concerning trends, the sponsor will meet with the site and remediation plan will be requested.

#### Chart re-abstraction

The site research team will re-abstract a subsample of their hospital charts and assess the error rate. Re-abstraction will focus on critical data elements related to the primary and secondary objectives of the protocol. The number of charts to be re-abstracted for each 6-month interval will be based on the number of patients who enroll in the study during the 6-month period at each site as shown in Table 5.

**Table 5:** Chart re-abstraction

No. of patients enrolled in a 6-month period	No. of charts to be re-abstracted
0	0
1–14	1
15–24	2
25–34	3
35–44	4
45–54	5
55–64	6

The DCC will provide sites with the randomly selected subject IDs for re-abstraction. The site research team will identify an independent site Quality Control (QC) abstractor who will re-abstract and enter data into the EDC only for the QC process and will not abstract study data while QC activities are taking place. The DCC will generate a discrepancy report comparing study data abstracted by the site with the source information abstracted by the independent QC abstractor. The site manager will hold a QC Review Meeting with the independent site QC abstractor, study coordinator, and site abstractor(s) to review the discrepancies and identify errors. Together they will discuss and document the corrective action for each error identified. The DCC will create manual queries in the EDC to make any necessary corrections to the data that were identified during the QC Review Meeting. The research team will provide hospitals that have an error rate above the predefined threshold (five errors per review) with additional training, a hospital-specific assessment of the data collection process, and suggestions for process improvement. The research team will track hospitals by their error rates. The research team will share practices of those hospitals with exceptionally low error rates with hospitals working to improve their own process. The trial subcommittee will review error rates and re-abstraction data during monthly team calls. If errors exceed five errors per review, on two consecutive reviews, a remediation plan will be requested and shared with the study sponsor.

#### Site monitoring visits

Concerning trends identified through centralized monitoring and/or re-abstraction may prompt site monitoring visits. Staff from the Coordinating Center and NIH/NICHD personnel will visit the site(s) with concerning trends in order to ensure data quality and regulatory compliance and to evaluate the performance of site investigators and staff. Site monitoring visits will be structured and planned in advance. They will involve onsite review and inspection of study participant charts, essential documents, and research staff qualifications and responsibilities. It may also include direct observation of study procedures and protocol implementation, as well as inspection of facilities and pharmacies and interviews with key stakeholders and senior leadership at the sites. If pandemic-related travel restrictions remain in place, such site monitoring visits may also be conducted virtually.

### Plans for communicating important protocol amendments to relevant parties (e.g., trial participants, ethical committees) {25}

Proposed protocol and patient-facing material modifications are communicated to the Data Coordinating and Operations Center (DCOC) at University of Arkansas for Medical Sciences (UAMS) for submission to the cIRB at UAMS. Upon cIRB approval of the modification, the DCOC will send the amendment with an accompanying approval letter to sites via email, using a study-wide contact list. For changes to non-patient-facing material, the RTI DCC will update the study documents and send updated materials with an accompanying technical memo to sites via email, using a study-wide contact list. All final, updated documents will be posted to the study website hosted by the ISPCTN

### Dissemination plans {31a}

The protocol study team will make every attempt to publish results in peer-reviewed journals. The team will submit all final peer-reviewed journal manuscripts from this study to the digital archive PubMed Central upon acceptance for publication.

Deposit data for data sharing with other researchers. Within the bounds of relevant IRB approvals and guidelines for protection of personally identifiable data, the protocol study team will deposit de-identified data from this study in an appropriate, NIH-approved data repository.

## Discussion

Not applicable: We have no practical or operational issues to report that involve performing the study .

## Trial status

This protocol is currently on version 8, July 28, 2021. Trial enrollment began on September 8, 2020. The anticipated end of enrollment is December 31, 2023. Long-term follow-up will be completed on February 28, 2026. The trial was registered on January 2, 2020; NCT04214834.

## Supplementary Information

**Additional file 1.** Study Schedule of Activities.

## Data Availability

This study intends to comply and adhere to all NIH Data Sharing Policy and Policy on the Dissemination of NIH-Funded Clinical Trial Information.

## Abbreviations

ACT NOWS: Advancing clinical trials in neonatal opioid withdrawal syndrome
AE: Adverse event
Bayley-4: Bayley Scales of Infant and Toddler Development, Fourth Edition
BITSEA: Brief Infant Toddler Social Emotional Assessment
BMI: Body mass index
BPM: Beats per minute
CI: Confidence interval
cIRB: Central IRB
COI: Conflicts of interest
CQ: Caregiver questionnaire
DCC: Data Coordinating Center
DCOC: Data Coordinating and Operations Center
DSMC: Data Monitoring and Safety Committee
ECHO: Environmental influences on Child Health Outcomes
EDC: Electronic Data Capture
EEG: Electroencephalogram
ER: Emergency room
FCOI: Financial conflicts of interest
GEE: Generalized estimating equations
GLMM: Generalized linear mixed-effects model
HC: Head circumference
HEAL Initiative®: Helping to End Addiction Long-term Initiative®
HIPAA: Health Insurance Portability and Accountability Act
ICC: Intraclass correlation
ICMJE: International Committee of Medical Journal Editors
IDeA: Institutional Development Awards
IRB: Institutional Review Board
ISPCTN: IDeA States Pediatric Clinical Trials Network
IV: Intravenous
LOT: Length of treatment days
MASQ: Mood and Anxiety Questionnaire
MOP: Manual of Operations
MPAQ: Measures, the Maternal Postnatal Attachment Questionnaire
MRN: medical record number
NAS: Neonatal abstinence syndrome
NCATS: National Center for Advancing Translational Sciences
NICHD: Eunice Kennedy Shriver National Institute of Child Health and Human Development
NICU: Neonatal Intensive Care Unit
NIH: National Institutes for Health
NNNS: NICU Network Neurobehavioral Scale
NOWS: Neonatal opioid withdrawal syndrome
NRN: Neonatal Research Network
OR: Odds ratio
PI: Principal Investigator
PROMIS: Parent-Reported Outcome Measure Information System
QC: Quality control
SAE: Serious adverse event
SD: Standard deviation
U.S.: United States
UAMS: University of Arkansas for Medical Sciences
WHO: World Health Organization
