# Supplementary material for: Pragmatic, randomized, blinded trial to shorten pharmacologic treatment of newborns with neonatal opioid withdrawal syndrome (NOWS)
Source: Trials. 2023 Jul 21;24:466. doi: 10.1186/s13063-023-07378-x (PMC10362592; doi:10.1186/s13063-023-07378-x)
Supplement: Supplementary file 1 — Additional file 1. Study Schedule of Activities. [file 13063_2023_7378_MOESM1_ESM.docx]

# Study Schedule of Activities

| **Activity/Event** | **Study Time Period** | | | | | | | | | | | | | |
| --- | --- | --- | --- | --- | --- | --- | --- | --- | --- | --- | --- | --- | --- | --- |
|  | **Prior to Birth** | **Prior to Randomization** | | | **Randomization** | **Initiation of Study Intervention/Weaning** | **Completion of Study Intervention/Weaning** | **Post Intervention Evaluation** | **Hospital Discharge** | **1 Month Post Discharge*** | **6 months of age*** | **12 months of age*** | **18 months of age*** | **24 months of age*** |
|  |  | **At Risk for NOWS** | **Initiation of Pharmacological Treatment** | **Stabilization (opioid**  **dose that controls symptoms)** |  |  |  |  |  |  |  |  |  |  |
| Prenatal consultation | X |  |  |  |  |  |  |  |  |  |  |  |  |  |
| In-utero opioid exposure | X | X |  |  |  |  |  |  |  |  |  |  |  |  |
| NOWS Scoring |  | X |  |  |  |  |  |  |  |  |  |  |  |  |
| Non-pharmacologic bundle |  | X |  |  |  |  |  |  |  |  |  |  |  |  |
| NOWS symptoms present |  | X |  |  |  |  |  |  |  |  |  |  |  |  |
| Morphine or methadone treatment initiated |  |  | X |  |  |  |  |  |  |  |  |  |  |  |
| Screening |  |  | X |  |  |  |  |  |  |  |  |  |  |  |
| Start of stabilization dose |  |  |  | X |  |  |  |  |  |  |  |  |  |  |
| Consent | X | X | X |  |  |  |  |  |  |  |  |  |  |  |
| Enrollment |  |  |  | X |  |  |  |  |  |  |  |  |  |  |
| Baseline data collection (includes maternal and infant medial history, infant measurements at birth, etc.) |  |  | | | X |  |  |  |  |  |  |  |  |  |
| Eligibility confirmed, stabilization dose tolerated |  |  | | | X |  |  |  |  |  |  |  |  |  |
| Wean morphine or methadone |  |  | | |  | X |  |  |  |  |  |  |  |  |
| Monitoring of serious adverse events |  |  | | |  | X | X |  |  |  |  |  |  |  |
| Intervention data collection (includes information on primary drug dose, second or third line drugs, etc.) |  |  | | |  | X | X |  |  |  |  |  |  |  |
| NNNS assessment (24-48 hours following study drug cessation) |  |  | | |  |  |  | X |  |  |  |  |  |  |
| Discharge data collection (includes discharge/transfer/death) |  |  | | |  |  |  |  | X |  |  |  |  |  |
| Patient-Reported Outcomes Measurement Information System (PROMIS) Measurement of Caregiver Well-Being |  |  | | |  |  |  |  |  | X |  |  |  | X |
| Maternal Postnatal Attachment Questionnaire (MPAQ) |  |  | | |  |  |  |  |  | X |  |  |  |  |
| Infant weight, length, head circumference |  |  | | |  |  |  |  | X |  |  |  |  | X |
| Caregiver Questionnaire (CQ) (enteral feeds, acute/urgent care and/or ER visits and readmissions) |  |  | | |  |  |  |  |  | X | X | X | X | X |
| Death |  |  | | |  |  |  |  |  |  | X | X | X | X |
| Bayley Scales of Infant and Toddler Development, Fourth Edition (Bayley-4): Cognitive, Language, Motor, Social-Emotional, Adaptive Behavior |  |  | | |  |  |  |  |  |  |  |  |  | X |
| Brief Infant-Toddler Social and Emotional Assessment (BITSEA) |  |  | | |  |  |  |  |  |  |  |  |  | X |
| Contact information update |  |  | | |  |  |  |  | X | X | X | X | X |  |
